# Extracting Features of Active Transition Metal Electrodes for NO Electroreduction with Catalytic Matrices

**DOI:** 10.1021/acsami.3c03385

**Published:** 2023-04-25

**Authors:** Eleonora Romeo, María Fernanda Lezana-Muralles, Francesc Illas, Federico Calle-Vallejo

**Affiliations:** † Department de Ciència de Materials i Química Física & Institut de Química Teòrica i Computational (IQTCUB), Universitat de Barcelona, C/Martí i Franquès 1, Barcelona 08028, Spain; ‡ Nano-Bio Spectroscopy Group and European Theoretical Spectroscopy Facility (ETSF), Department of Polymers and Advanced Materials: Physics, Chemistry and Technology, University of the Basque Country UPV/EHU, Avenida Tolosa 72, San Sebastián 20018, Spain; § IKERBASQUE, Basque Foundation for Science, Plaza de Euskadi 5, Bilbao 48009, Spain

**Keywords:** nitric oxide reduction, nitric oxide hydrogenation, electrocatalysis, structural sensitivity, reaction mechanism

## Abstract

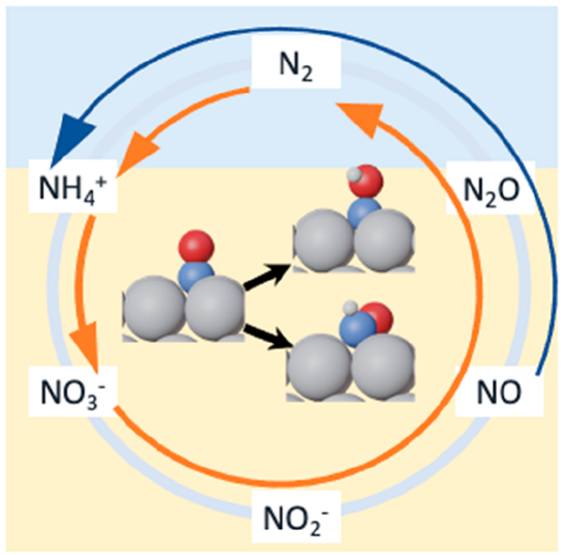

Electrocatalytic reduction of oxidized nitrogen compounds (NO_*x*_) promises to help rebalance the nitrogen cycle. It is widely accepted that nitrate reduction to NH_4_^+^/NH_3_ involves NO as an intermediate, and NO hydrogenation is the potential-limiting step of NO reduction. Whether *NO hydrogenates to *NHO or *NOH is still a matter of debate, which makes it difficult to optimize catalysts for NO_*x*_ electroreduction. Here, “*catalytic matrices*” are used to swiftly extract features of active transition metal catalysts for NO electroreduction. The matrices show that active catalysts statistically stabilize *NHO over *NOH and have undercoordinated sites. Besides, square-symmetry active sites with Cu and other elements may prove active for NO electroreduction. Finally, multivariate regressions are able to reproduce the main features found by the matrices, which opens the door for more sophisticated machine-learning studies. In sum, catalytic matrices may ease the analysis of complex electrocatalytic reactions on multifaceted materials.

## 1 Introduction

Nitrogen is the most abundant element in the Earth’s atmosphere, and various N-containing compounds are created and consumed in nature as part of a dynamic but delicate environmental equilibrium known as the nitrogen cycle. In a well-balanced nitrogen cycle, several biogeochemical processes successively transform atmospheric N_2_ into NH_4_^+^, NO_2_^–^, and NO_3_^–^ and back to N_2_. Specifically, nitrogen fixation in the soil in the form of NH_3_/NH_4_^+^ originates from organic matter in decomposition, bacteria, and plants. In addition, natural nitrification processes occur in the soil that transform ammonia into nitrite and nitrate, and then denitrification bacteria or plants reduce the latter and release N_2_ back to the atmosphere, closing the cycle.^1,2^

Importantly, the Haber-Bosch synthesis (N_2_ + 3H_2_ → 2NH_3_),^3,4^ provided the basis for the massive production of synthetic fertilizers, which ultimately facilitated an unprecedented growth of the human population.^5^ The Haber-Bosch synthesis is among the most energy-intensive industrial processes, requiring active Fe-based catalysts and high temperatures and pressures (400–500 °C, 100–300 bar),^6^ and the H_2_ needed for the reaction is usually obtained from fossil fuels via steam reforming of natural gas.^7−9^ Apart from that, the widespread use of fertilizers causes the excessive release of nitrate to the soil, which resulted in a tremendous imbalance of the nitrogen cycle.^10,11^ In fact, ground and surface waters currently display alarming concentrations of nitrate and nitrite, which have presumably created dead zones in coastal areas^12^ and may lead to severe health issues.^13,14^

Denitrification of NO_*x*_ compounds in electrolyzers is a promising strategy to help rebalance the nitrogen cycle. Indeed, N_2_, N_2_O, NH_3_, and NH_2_OH under mild operating conditions can be produced using electricity from renewable sources.^15,16^ However, numerous challenges hamper the design of efficient and selective electrocatalysts made upon abundant elements. One such challenge is the elaboration of a comprehensive reaction mechanism that incorporates factors such as pH, applied potential, and structural sensitivity.

It is widely accepted that during NO_3_^–^ reduction to NH_3_/NH_4_^+^, NO is formed.^17^ Besides, NO hydrogenation is the potential-limiting step of NO reduction and is key to regulating the selectivity of NO_*x*_ electroreduction.^17−20^ Importantly, it is not yet obvious when *NO hydrogenation leads to *NHO or *NOH and various opinions are found in the literature. For example, using DFT calculations and classification methods, Wan et al. investigated the NO_*x*_ reduction selectivity and activity of a series of metal electrodes.^21^ They found that various Cu facets are selective to NH_3_ by virtue of their moderate adsorption energies of *NO and *H. They further suggested *NHO as a hydrogenation intermediate for Ag and Au, whereas *NOH is formed on Cu. Moreover, Casey-Stevens et al.^18^ explored possible mechanisms toward the formation of NH_4_^+^, NH_3_OH^+^, and N_2_O from NO electroreduction on various transition metal electrodes using DFT calculations. They found that *NOH formation is the potential-limiting step for NH_4_^+^ production on Cu(111), Rh(111), and Pd(111), while for Ag(111) and Au(111) it is *NHO formation.

Clayborne et al.^20^ combined ground-state and transition-state DFT calculations to inspect *NO electroreduction on Pt(111). They concluded that the path leading to NH_3_/NH_4_^+^ at low coverage of *NO involves *NOH. This adsorbate is also kinetically favored on Pt(100), as suggested by the agreement between the simulated and experimental reductive stripping voltammetry of NO.^22^ Katsounaros et al.^19^ combined experiments and DFT calculations to investigate the structure-sensitive electroreduction of NO on Pt. They showed that Pt(111) and Pt(100) exhibit different behavior: at low coverage (0.25 ML *NO), Pt(111) hydrogenates *NO to *NOH. When the *NO coverage is 0.50 ML, *NO is initially reduced to *NHO, and then to *NOH. On Pt(100), at high *NO coverage (0.50 ML *NO), one-half of the *NO is hydrogenated to *NOH, and the other half to *NHO.

In perspective, all these observations suggest that *NO hydrogenation to *NOH or *NHO depends not only on the type of metal but also on additional factors such as the geometry of the surface sites and their availability. Hence, a fair question is whether the structural sensitivity of active NO electroreduction catalysts can be established in simple terms. To address this point, here we present “catalytic matrices”, which help to rapidly examine the structure-sensitive activity and selectivity trends for electrocatalytic *NO hydrogenation on six active sites of various surfaces of nine transition metals. Catalytic matrices enable a statistical treatment of the trends that leads to qualitative and quantitative conclusions. In particular, the matrices show that active catalysts for *NO electroreduction ought to contain undercoordinated Cu sites, and active alloys may be formed that contain square-symmetry sites. Finally, we supplement our study by showing that the matrices are more descriptive than adsorption-energy scaling relations and that multivariate regressions can reproduce their main features, which opens the door for future studies with more advanced machine learning techniques.

## 2 Computational Details

We simulated slabs of Co, Ni, Cu, Rh, Pd, Ag, Ir, Pt, and Au. For each metal, we explored the (111), (100), and (211) facets. Besides, we created a kink on the (211) surface, denoted (211)k, and two types of metal adatom islands: 4AD@(100), which contains 4-atom islands on top of (100) terraces, and 3AD@(111), which contains 3-atom islands on top of (111) terraces. These surfaces contain sites with different coordination numbers (*cn*): 9, 8, 7, 6, 6, and 5 for (111), (100), (211), (211)k, 4AD@(100), and 3AD@(111), respectively. Additional details on the slabs and their adsorption sites are provided in Tables S1 and Figures S1 and S2.

The DFT calculations were carried out with the VASP code.^23^ The Perdew–Burke–Ernzerhof (PBE) exchange-correlation functional^24^ was chosen because it accurately describes the three series of transition metals^25,26^ and their low Miller index surfaces.^27^ The valence electron density was expanded using a plane-wave basis set with a cutoff of 450 eV for the kinetic energy. The effect of the atomic cores on the valence electron density was incorporated by means of the projector augmented-wave method.^28^ The Methfessel-Paxton approach^29^ was used to smear the Fermi level with *k*_B_*T* = 0.2 eV, always extrapolating the total energies to 0 K upon convergence. The calculations were spin unrestricted for Co and Ni slabs and gas-phase NO. The numerical integration in the reciprocal space was carried out using Monkhorst–Pack^30^ grids of special ***k***-points. To avoid spurious electrostatic interactions between periodically repeated slabs, periodic images in the vertical direction were separated by more than 15 Å of vacuum and dipole corrections were also applied. The conjugate gradient algorithm was used for the geometry optimizations, with iterations performed until the maximal force on all atoms was below 0.05 eV Å^–1^. The adsorbates, adatom islands, and the top two metal layers of the slabs were relaxed in all directions, while the bottom layers were fixed at the bulk equilibrium distances to provide an adequate environment below the surface region. Boxes of 9 × 10 × 11 Å^3^ were used to calculate O_2_, H_2_, H_2_O, N_2_, NH_3_, and NO, considering the Γ-point only, using Gaussian smearing and *k*_B_*T* = 0.001 eV, with further extrapolation to 0 K.

The Gibbs free energy difference for the considered elementary steps (Δ*G*) was approximated as Δ*G* ≈ Δ*E*_DFT_ + ΔZPE – *T*Δ*S* + Δ*E*_solvation_, where Δ*E*_DFT_ is the PBE-calculated energy difference, ΔZPE is the zero-point energy change, and *T*Δ*S* is the corresponding entropy change at 298.15 K. For gas-phase molecules the total entropies were obtained from thermodynamic tables,^31^ and their free energies were corrected semiempirically (more details on this procedure are available in section S1),^32−35^ while for adsorbates Δ*S* only includes vibrational entropies. Δ*E*_solvation_ is the contribution of solvent-adsorbate interactions to the free energy, which we evaluated using four different solvation approaches, see sections S1 and S2. The results in the figures and tables below are from an approach combining microsolvation^36^ and implicit solvation.^37^ Finally, the energetics of proton–electron pairs was described by means of the computational hydrogen electrode, which seizes the equilibrium in solution between those and gaseous hydrogen.^38^

## 3 Results and Discussion

**Figure 1:**
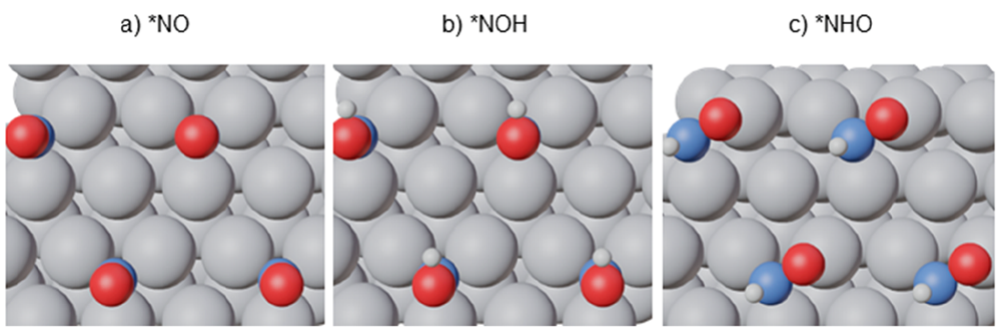
Top views of *NO, *NOH, and *NHO on Pt(111). *NO and *NOH are monodentate while *NHO is bidentate. Color code: Pt in gray, N in blue, O in red, H in white. See also Figure S4–S9.

Electrocatalytic hydrogenation of *NO in acid media involves a proton–electron transfer and two possible products can be formed:
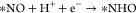
1
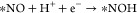
2*NO and *NOH are monodentate adsorbates bound to the surface by the N atom, while *NHO is generally a bidentate adsorbate bound via N and O. To illustrate this, Figure 1 provides top views of *NO, *NOH and *NHO on Pt(111). The corresponding side views together with images for the rest of surfaces are found in Figures S4–S9.

Table 1 is a structure-sensitive matrix for the selectivity of *NO hydrogenation on late transition metal surfaces. The matrix shows on a per metal and facet basis the cases where *NOH, *NHO or both are the most stable products of *NO hydrogenation. Since the accuracy of DFT-PBE calculations is around ±0.1 eV, we consider *NOH and *NHO to be similarly stable when: *abs*(Δ*G*_NHO_ – Δ*G*_NOH_) < 0.1 eV. In such case, both hydrogenated intermediates are likely to be formed and transition-state searches are advisable.^20^ We note that Table 1 contains data with a mixed solvation approach^36,37^ and analogous tables for data in a vacuum and with the remaining solvation methods are reported in Tables S2–S5.

**Table 1:**
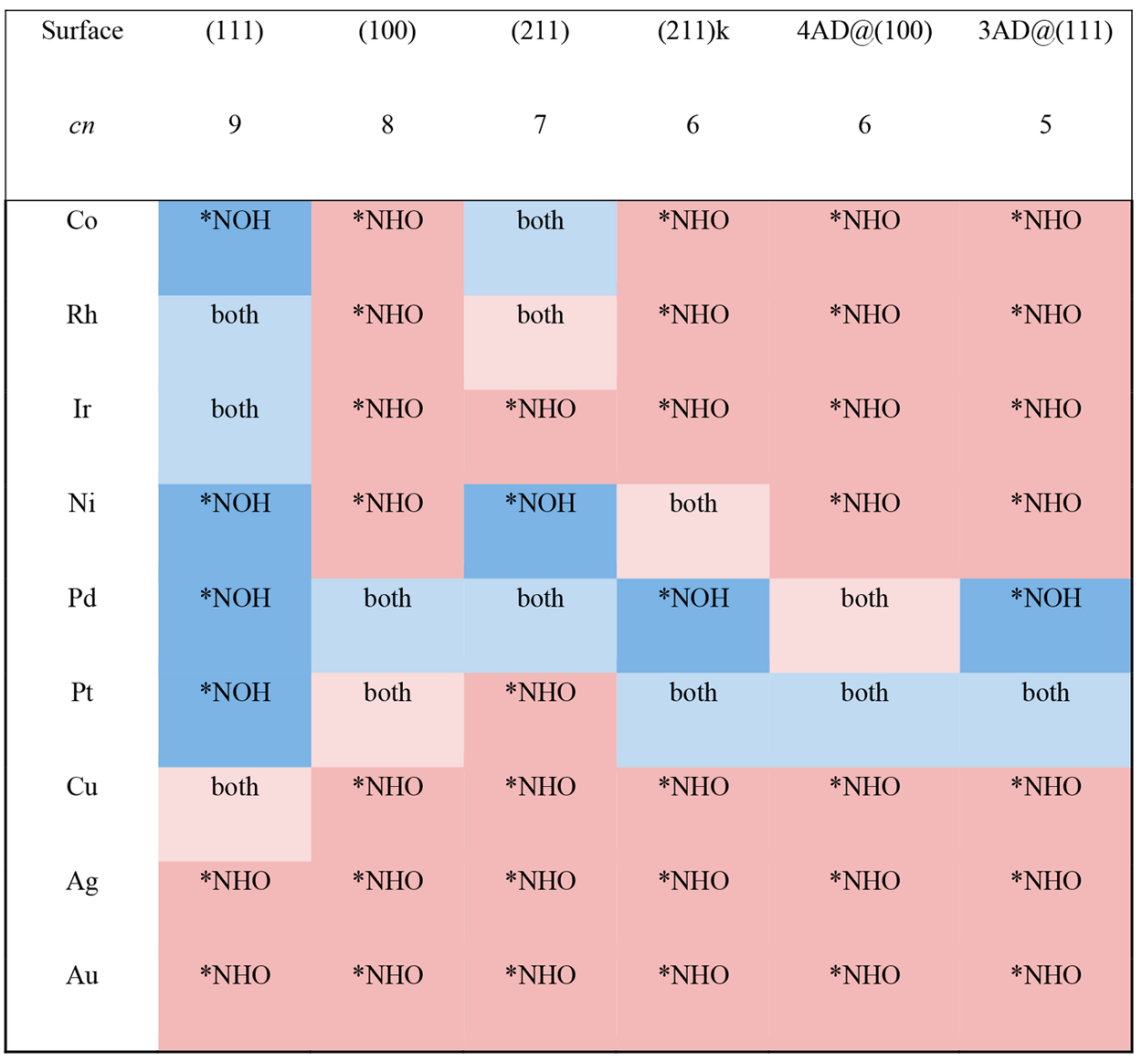
Structure-Sensitive Selectivity Matrix for *NO Hydrogenation on Transition Metals^a^

a *cn*: coordination number of the active sites. Color code: blue for *NOH, red for *NHO; light blue/red indicates both adsorbates might be formed but with a slight tendency toward *NOH/*NHO, such that *abs*(Δ*G*_NHO_ – Δ*G*_NOH_) < 0.1 eV.

The selectivity matrix can be analyzed in terms of its rows (metals) and columns (active sites), such that it is both material- and structure-sensitive. According to the rows, *NHO dominates on Cu, Ag, and Au (group 11 of the periodic table). *NOH or both are common on Ni, Pd, and Pt (group 10). On Co, Rh, and Ir (group 9) the most common product is again *NHO, particularly when the adsorption sites are undercoordinated and/or have square symmetry. In turn, the columns of the selectivity matrix indicate a clear difference among (111) terraces and the other surface sites: most facets are statistically inclined toward *NHO except for (111) terraces. This means that Cu, Ag, Au, and Pd polycrystalline electrodes would be well represented by the (111) facet, given that the other ones exhibit similar selectivity; Pt electrodes are represented by Pt(111) only to a certain extent and there are noticeable differences between the (111) and (100) facets, in line with experiments;^19^ and Co, Rh, Ir, and Ni electrodes are not well represented by their (111) surfaces. This suggests that the widespread use of the (111) surface as a representation of an entire catalyst might be problematic for various polycrystalline transition metal electrodes.

**Figure 2:**
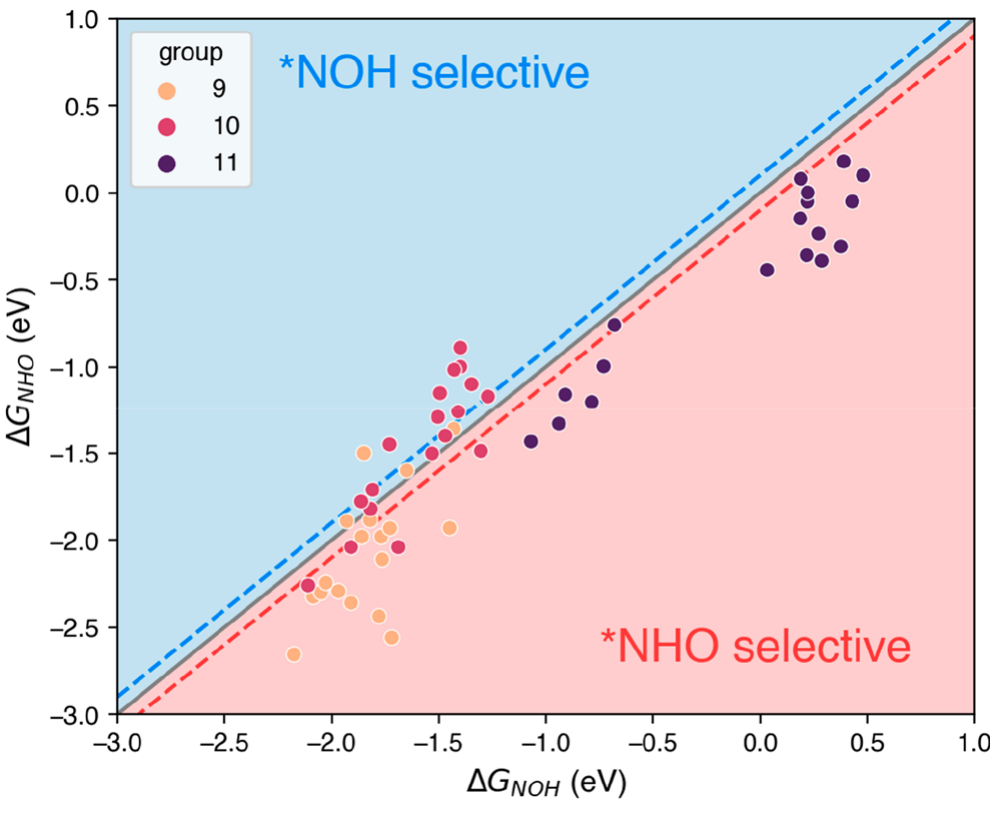
Parity plot for Δ*G*_NHO_ vs Δ*G*_NOH_ for all analyzed metals and surfaces. Group 11 metals (Cu, Ag and Au; in purple) prefer *NHO, hence their location below the parity line. Numerous sites on group 10 metals (Ni, Pd, Pt; in red) are selective to *NOH, particularly for weak binding energies. Group 9 metals (Co, Rh, Ir; in orange) mostly favor *NHO formation, especially for strong binding energies. In the region within the dashed blue/red lines (where *abs*(Δ*G*_NHO_–Δ*G*_NOH_) < 0.1 eV) both adsorbates might form. See also Tables 1, 2, and S6–S8.

For comparison, the selectivity trends are presented in Figure 2 as a parity plot. In it, the adsorption energies of the two competing adsorbates of *NO hydrogenation are plotted as a scaling relation.^39^ Data points above the parity line (where Δ*G*_NHO_ = Δ*G*_NOH_) represent sites inclined to produce *NOH, and the opposite is true for sites producing *NHO. The selectivity matrix in Table 1 is more illustrative of the trends, as it shows that transition metals within a given group of the periodic table and also certain active sites tend to behave similarly. The difficulties extracting overall features from a large data set comprising numerous facets by means of scaling relations is exemplified in section S8, where a comprehensive analysis is provided. Our conclusion is that a matrix representation of the trends is, in this case, a more efficient way of condensing and analyzing large amounts of data.

Table 2 summarizes the percentage of cases where *NOH, *NHO, or both, are the most stable intermediates. In general, *NHO seems to be the main product across the different surface sites among the nine metals analyzed here. In fact, nearly two-thirds of the active sites are predicted to produce *NHO from *NO hydrogenation. However, Table 2 shows that the selectivity varies from one group of the periodic table to another: *NHO formation is favored by Cu, Ag, Au (group 11), and Co, Rh, and Ir (group 9), while Ni, Pd, and Pt (group 10) have mixed selectivity or are inclined toward *NOH.

**Table 2:**
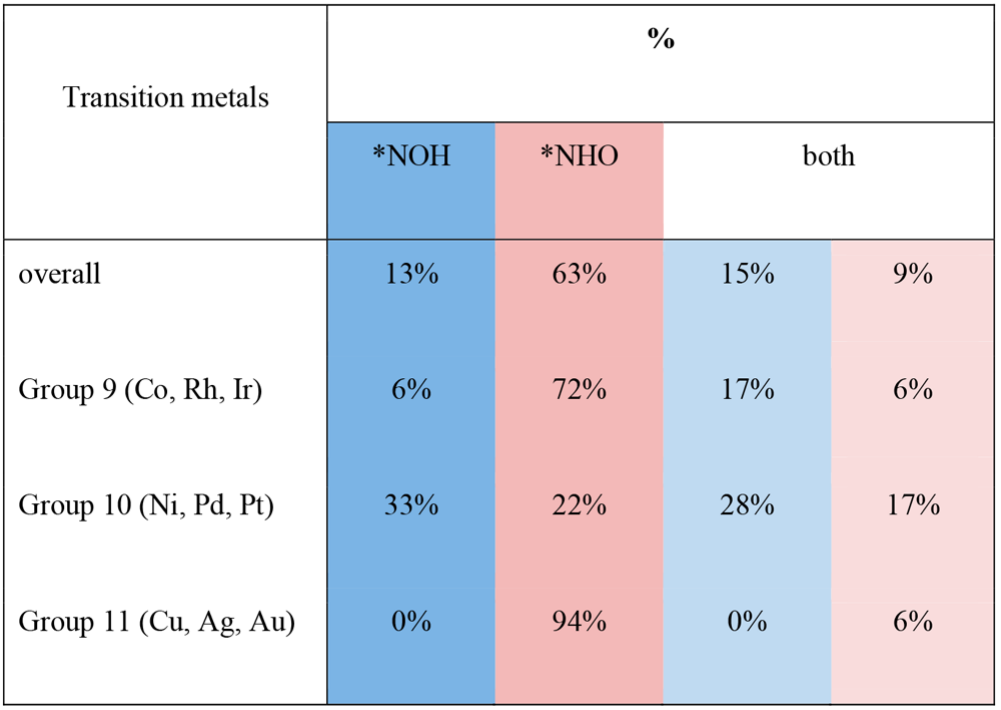
Selectivity of *NO Hydrogenation^a^

a The percentages indicate the fraction of sites that prefer a given intermediate (*NOH, *NHO or both) in Table 1 with respect to the total number of sites. Light blue/red indicates that both adsorbates might be formed but lean toward *NOH/*NHO, such that *abs*(Δ*G*_NHO_ – Δ*G*_NOH_) < 0.1 eV). The overall percentage for *NHO is generally higher, given that elements in groups 9 and 11 favor its formation, while elements in group 10 favor *NOH.

We note in passing that the results in Tables 1, 2, and S2–S8 stress the importance of accounting for solvent-adsorbate interactions but show that the different methods provide similar conclusions. However, the choice of solvation method has proved crucial in quantitative analyses aimed at comparing with experiments, such as the assessment of catalytic pathways and onset potentials for other reactions.^36,40−42^

Often, the potential-limiting step of NO reduction to NH_4_^+^ and hydroxylamine on transition metals is the hydrogenation of *NO to *NHO or *NOH.^18−20^ Thus, this step may as well be important for the activity and dictates the onset potential of the overall NO reduction reaction. While the equilibrium potential for NO reduction to NH_4_^+^ is as high as 0.84 V vs RHE (reversible hydrogen electrode; hereon the potentials are reported in that scale), the onset potentials of transition metals are usually in the range of 0.0 to 0.3 V.^18−20,43^ This implies that the overpotentials for NO reduction are at least 0.5 V and, in consequence, *NO hydrogenation might be as endothermic as 0.5 eV or more at the equilibrium potential. In this order of ideas, *NO hydrogenation can be used as a proxy to evaluate the activity of the entire catalytic pathway, and promising active sites can be the subject of further, more comprehensive studies, including kinetic analyses and competing reactions such as hydrogen evolution.^20−22^

Based on the potential required for *NO hydrogenation, we provide in Table 3 an activity matrix for NO reduction where *U*_onset_ is estimated as
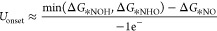
3The matrix helps distinguish between very active (*U*_onset_ > 0.3 V), active (−0.05 V < *U*_onset_ < 0.3 V), and inactive sites (*U*_onset_ < – 0.05 V), see further details in section S3. Interestingly, the rows of Table 3 show that the (100) terraces of most transition metals tend to be active for *NO hydrogenation, and that most facets of group 11 metals are also active, particularly those of Cu, which is known in experiments to be considerably active for NO_3_^–^ reduction to NH_3_/NH_4_^+^ via *NO.^44,45^ Besides, the activity seems to rapidly decrease from group 11 to groups 10 and 9 of the periodic table. We note that *U*_onset_ is a thermodynamic descriptor. When used to make activity predictions, it is assumed that reaction thermodynamics and kinetics are proportional throughout the reaction pathway. In particular, the potential-limiting step (PLS) and the rate-determining step (RDS) should either coincide or at least be modified by the applied potential in the same way.^46^ This and other assumptions and simplifications have recently been analyzed by Razzaq and Exner in the light of the free-energy span model.^47^

**Table 3:**
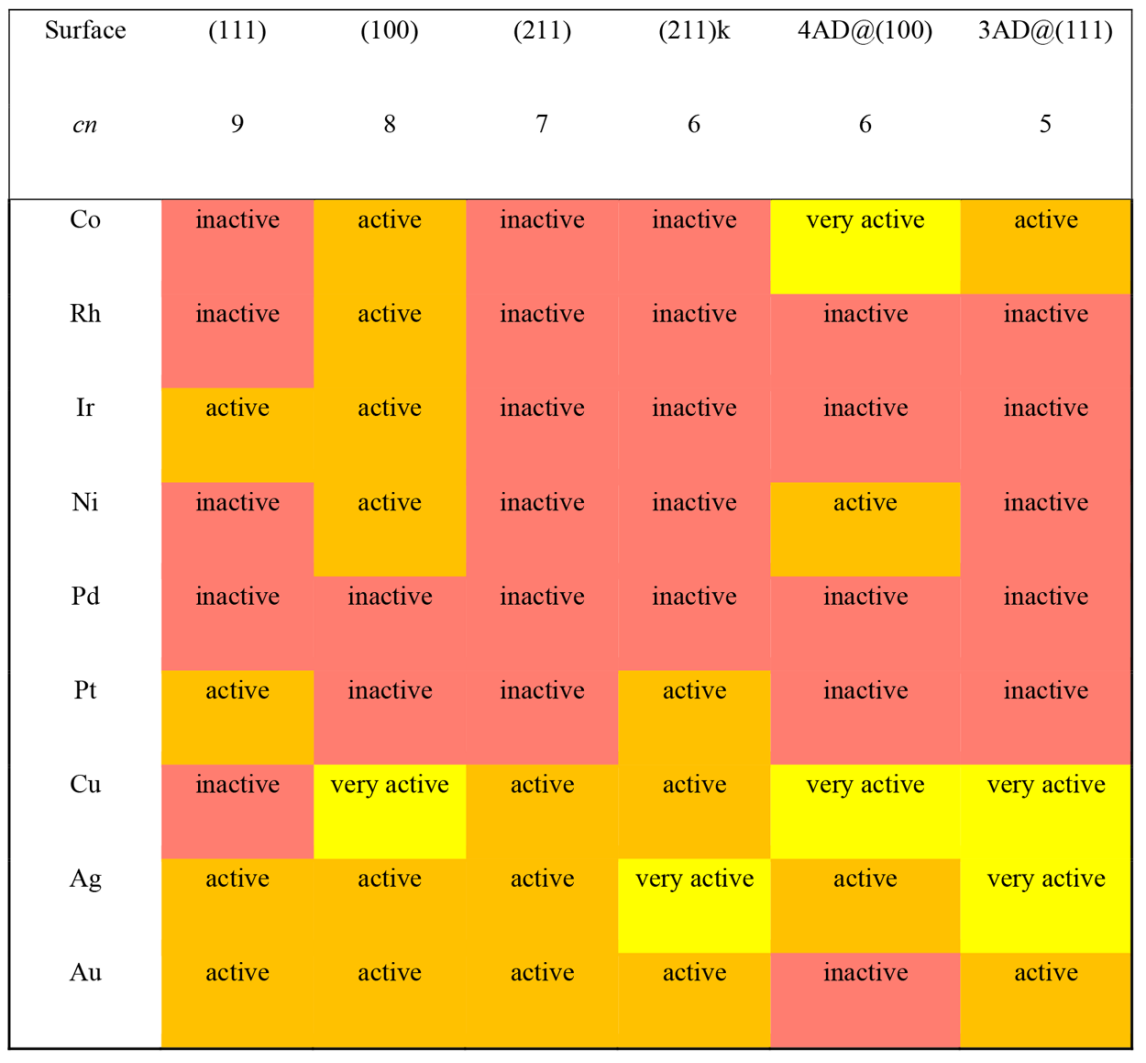
Activity Matrix for *NO Hydrogenation^a^

a The matrix classifies the sites as very active (*U*_onset_ > 0.3 V, yellow), active (−0.05 V < *U*_onset_ < 0.3 *V*, orange) and inactive (*U*_onset_ < −0.05 V, red). *cn*: coordination number of the active sites.

It is possible to mimic the distinction between active and inactive sites in Table 3 by means of multivariate linear regressions, which are a supervised-type of machine learning approach (see section S10). Initially, we used only three attributes of the active sites as input variables, namely *cn* and the group number and period of the metal in the periodic table. These three parameters are present in catalytic matrices, as they have coordination numbers in the columns and 4d, 5d, and 6d metals in the rows. For Pt(111), for instance, the values are 9, 10 and 6, respectively. As shown in Table S12, the algorithm predicts a catalytic matrix that distinguishes between active and inactive sites in 74% of the cases. To increase the accuracy of the predictions, we added an additional parameter not directly related to the onset potential, ΔG_*NHO_ – ΔG_*NOH_, which enables the model to correctly distinguish between active and inactive sites in 87% of the cases, see Table 4. We explain why we chose this energetic parameter, why it improves the predicted matrix and compare to other parameters in section S10. To further increase the activity and be able to distinguish between active and very active sites, it is probably necessary to add more parameters to the model and/or use advanced algorithms. However, these results are encouraging, as the main distinction between active and inactive sites can be replicated to a great extent by multivariate regressions using three basic and readily available attributes of active sites and an optional energetic parameter.

**Table 4:**
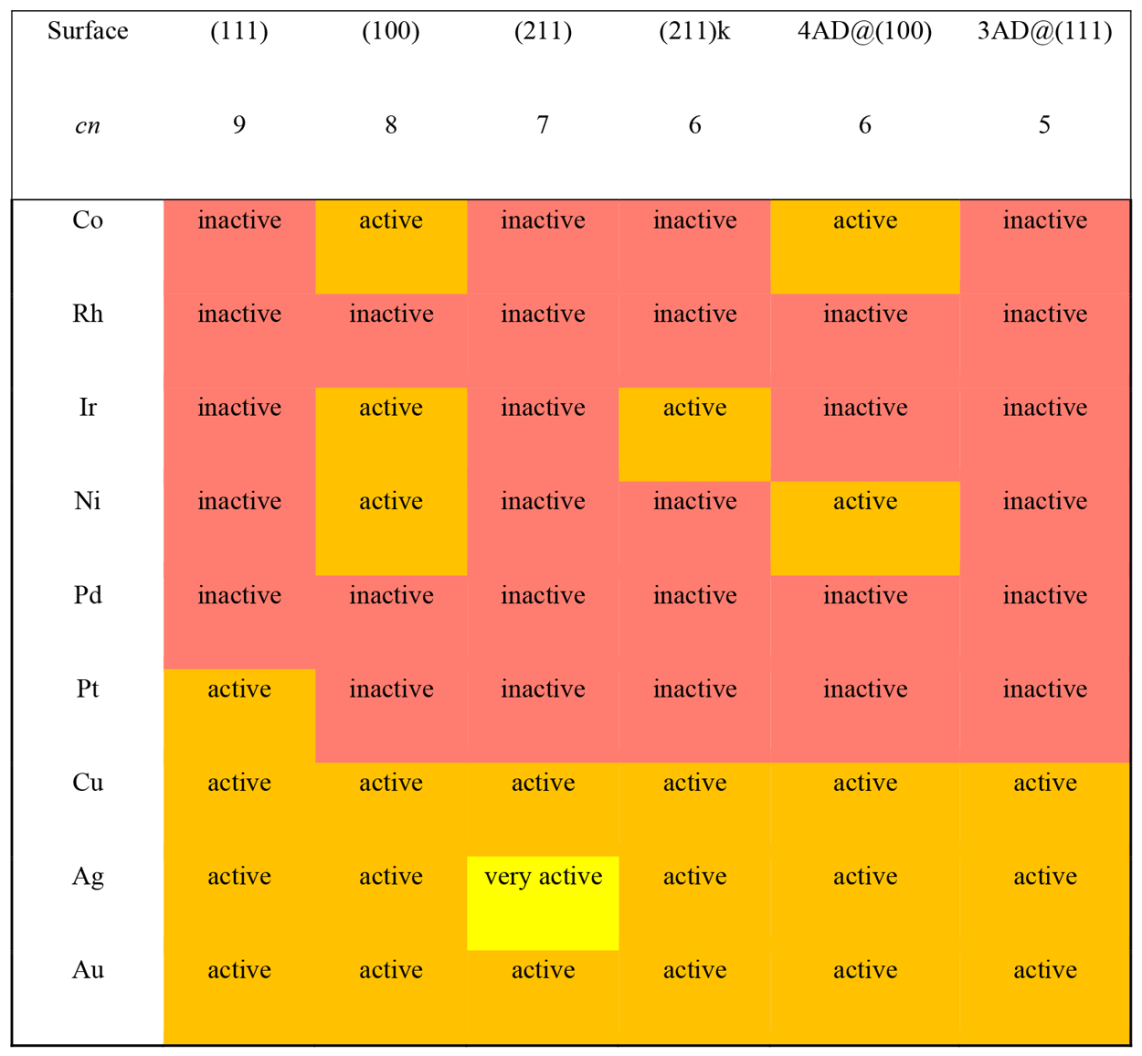
Predicted Activity Matrix for *NO Hydrogenation Using As Independent Variables *cn*, the Group and Period in the Periodic Table of the Metal, and Δ*G*_NHO_ – Δ*G*_NOH_^a^

a The sites are classified as very active (*U*_onset_ > 0.3 V, yellow), active (−0.05 V < *U*_onset_ < 0.3 V, orange), and inactive (*U*_onset_ < −0.05 V, red).

Finally, it is possible to combine the selectivity and activity matrices (Tables 1 and 3) to simultaneously observe the trends in activity and selectivity of *NO hydrogenation, as shown in Table 5. The activity-selectivity matrix tells whether or not specific metals and facets are active for *NO hydrogenation and via which intermediate. This is important to know in electrocatalysis, as usually a given adsorbate is held responsible for the poor catalytic performance for a given reaction: that is the case of *CHO for CO_2_ reduction to CH_4_,^48^ and the case of *OOH for oxygen reduction and evolution.^49,50^ We observe in Table 5 that group 11 metals are active for *NO hydrogenation via *NHO, and Cu(111) terraces are not active for *NO reduction at moderate overpotentials. Instead, most of the activity of Cu electrodes should come from undercoordinated sites.

**Table 5:**
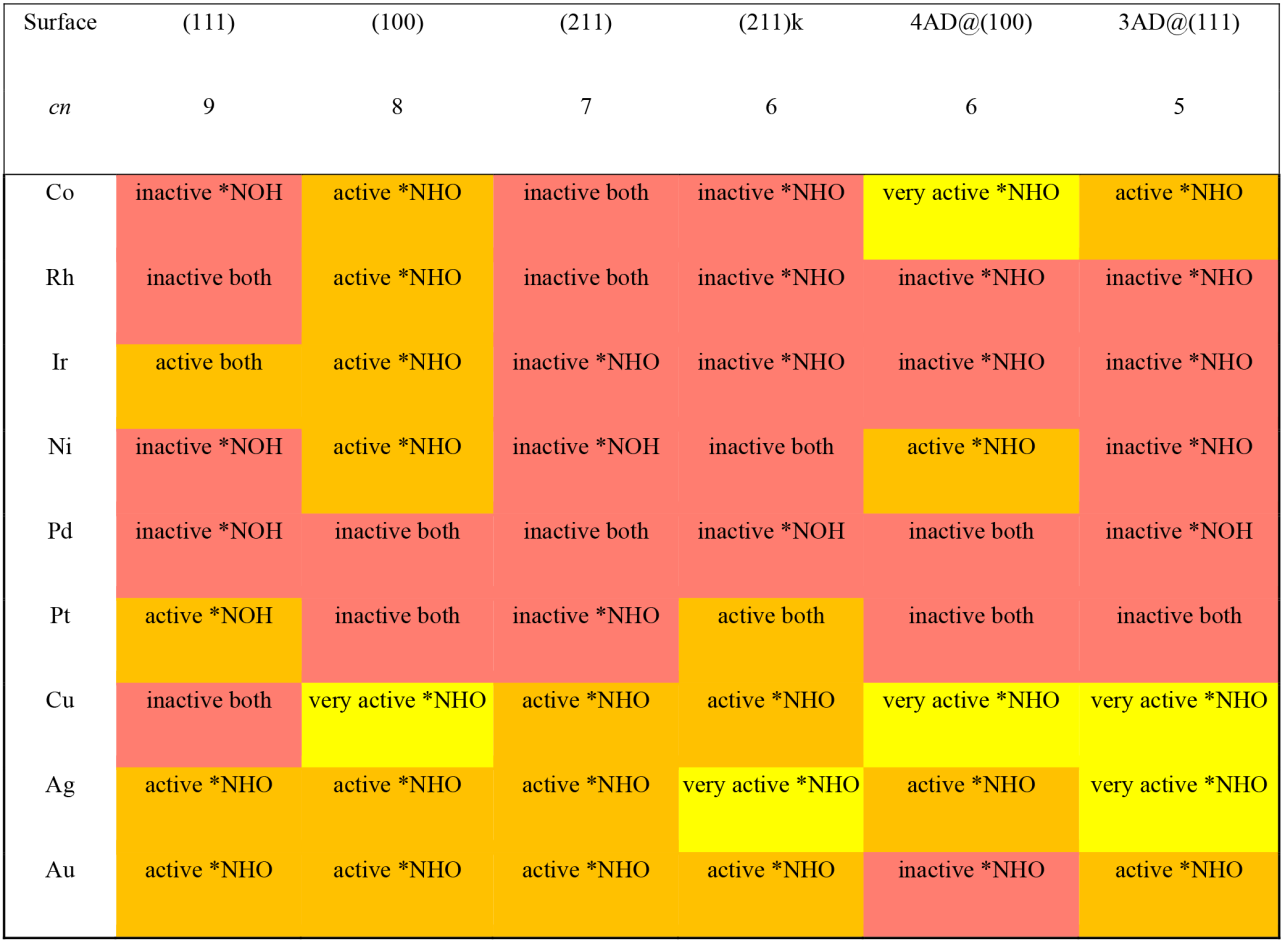
Activity/Selectivity Matrix for *NO Hydrogenation^a^

a The matrix classifies the sites as very active (*U*_onset_ > 0.3 V), active (−0.05 V ≤ *U*_onset_ ≤ 0.3 V), and inactive (*U*_onset_ < −0.05 V) and indicates in each case the most stable product of *NO hydrogenation. *cn*: coordination number of the surface atoms.

It is possible to make an overall assessment of transition metal electrodes for *NO hydrogenation. According to Table 5, among all the examined sites on transition metals, 52% are inactive as they need overpotentials larger than ∼0.90 V (i.e., *U*_onset_ < −0.05 V) for *NO hydrogenation to become exergonic. Sites classified as very active are 11% of the total and all of them are inclined toward *NHO. Active sites inclined to *NHO correspond to 31% of the cases, 2% of the active sites lean toward *NOH, and 4% may concurrently produce *NOH and *NHO. Interestingly, for sites classified as active or very active, the selectivity is clearly inclined toward *NHO (96%) compared to *NOH (4%). Before closing the discussion, we note that Cu–Ni alloys have been shown in experiments to be highly active for nitrate reduction via *NO reduction, but the structural sensitivity of the active sites was not inspected.^45^ Judging by the results in Table 5, we propose that the active Cu–Ni sites should have square symmetry and reduce *NO to *NHO.

## 4 Conclusions

Extracting general features of active and selective electrodes is a major challenge in electrocatalysis, where the interplay of pH, applied potential and structural sensitivity influences the intrinsic catalytic activity and selectivity of electrode materials. This is particularly true for reactions with numerous electron transfers and several products, as is the case of NO_*x*_ reduction. Here we showed that the structural sensitivity of *NO hydrogenation on multifaceted transition metal electrodes can be described by means of catalytic matrices, which provide insightful qualitative and quantitative information in a swift way. Numerous surfaces are inclined to produce *NHO, and the selectivity is divided into three groups: *NHO predominates on Cu, Ag, and Au (group 11); *NOH or both are formed on Ni, Pd and Pt (group 10); and on Co, Rh, and Ir (group 9) *NHO is mostly produced, especially when the adsorption sites are undercoordinated and/or have square symmetry. Furthermore, group 11 elements (especially Cu) tend to be active for *NO hydrogenation and likely for NO reduction to NH_4_^+^/NH_3_, while the (100) terraces of various transition metals should also be active. Multivariate regressions show that the features extracted from catalytic matrices can be machine-learned using basic features of the active sites, which opens the door for future studies.

Finally, activity-selectivity matrices indicate that active materials for NO reduction should usually contain undercoordinated Cu sites and be mediated by *NHO. This piece of information and others extracted from the catalytic matrices can be used to outline active sites that enhance materials for NO_*x*_ electroreduction.

## Supporting Information Available

Additional computational details, description of the microsolvation calculations and the activity assessment, additional selectivity matrices, tabulated values, and analyses based on adsorption-energy scaling relations (PDF)

## References

[ref1] McNeill A.; Unkovich M. The Nitrogen Cycle in Terrestrial Ecosystems. In Nutrient Cycling in Terrestrial Ecosystems; Marschner P., Rengel Z., Eds.; Soil Biology; Springer: Berlin, 2007; pp 37–64.

[ref2] Rosswall T. The Internal Nitrogen Cycle between Microorganisms, Vegetation and Soil. Ecol. Bull. 1976, 22, 157–167.

[ref3] Haber F. The Synthesis of Ammonia from Its Elements. In Nobel Lectures, Chemistry 1901–1921; Elsevier: Amsterdam, 1966.

[ref4] Bosch C. The Development of the Chemical High Pressure Method During the Establishment of the New Ammonia Industry. In Nobel Lecture, Chemistry 1922–1941; Elsevier: Amsterdam, 1966.

[ref5] Smil V. Detonator of the Population Explosion. Nature 1999, 400 (6743), 415–415. 10.1038/22672.

[ref6] Kandemir T.; Schuster M. E.; Senyshyn A.; Behrens M.; Schlögl R. The Haber–Bosch Process Revisited: On the Real Structure and Stability of “Ammonia Iron” under Working Conditions. Angew. Chem., Int. Ed. 2013, 52 (48), 12723–12726. 10.1002/anie.201305812. 24123652

[ref7] Van Hook J. P. Methane-Steam Reforming. Catal. Rev. 1980, 21 (1), 1–51. 10.1080/03602458008068059.

[ref8] Holladay J. D.; Hu J.; King D. L.; Wang Y. An Overview of Hydrogen Production Technologies. Catal. Today 2009, 139 (4), 244–260. 10.1016/j.cattod.2008.08.039.

[ref9] Smith C.; Hill A. K.; Torrente-Murciano L. Current and Future Role of Haber–Bosch Ammonia in a Carbon-Free Energy Landscape. Energy Environ. Sci. 2020, 13 (2), 331–344. 10.1039/C9EE02873K.

[ref10] Gruber N.; Galloway J. N. An Earth-System Perspective of the Global Nitrogen Cycle. Nature 2008, 451 (7176), 293–296. 10.1038/nature06592. 18202647

[ref11] Rockström J.; Steffen W.; Noone K.; Persson Å.; Chapin F. S.; Lambin E. F.; Lenton T. M.; Scheffer M.; Folke C.; Schellnhuber H. J.; Nykvist B.; de Wit C. A.; Hughes T.; van der Leeuw S.; Rodhe H.; Sörlin S.; Snyder P. K.; Costanza R.; Svedin U.; Falkenmark M.; Karlberg L.; Corell R. W.; Fabry V. J.; Hansen J.; Walker B.; Liverman D.; Richardson K.; Crutzen P.; Foley J. A. A Safe Operating Space for Humanity. Nature 2009, 461 (7263), 472–475. 10.1038/461472a. 19779433

[ref12] Diaz R. J.; Rosenberg R. Spreading Dead Zones and Consequences for Marine Ecosystems. Science 2008, 321 (5891), 926–929. 10.1126/science.1156401. 18703733

[ref13] Lundberg J. O.; Weitzberg E.; Cole J. A.; Benjamin N. Nitrate, Bacteria and Human Health. Nat. Rev. Microbiol. 2004, 2 (7), 593–602. 10.1038/nrmicro929. 15197394

[ref14] Pennino M. J.; Compton J. E.; Leibowitz S. G. Trends in Drinking Water Nitrate Violations Across the United States. Environ. Sci. Technol. 2017, 51 (22), 13450–13460. 10.1021/acs.est.7b04269. 29052975 PMC5764095

[ref15] van Langevelde P. H.; Katsounaros I.; Koper M. T. M. Electrocatalytic Nitrate Reduction for Sustainable Ammonia Production. Joule 2021, 5 (2), 290–294. 10.1016/j.joule.2020.12.025.

[ref16] Garcia-Segura S.; Lanzarini-Lopes M.; Hristovski K.; Westerhoff P. Electrocatalytic Reduction of Nitrate: Fundamentals to Full-Scale Water Treatment Applications. Appl. Catal. B Environ. 2018, 236, 546–568. 10.1016/j.apcatb.2018.05.041.

[ref17] Rosca V.; Duca M.; de Groot M. T.; Koper M. T. M. Nitrogen Cycle Electrocatalysis. Chem. Rev. 2009, 109 (6), 2209–2244. 10.1021/cr8003696. 19438198

[ref18] Casey-Stevens C. A.; Ásmundsson H.; Skúlason E.; Garden A. L. A Density Functional Theory Study of the Mechanism and Onset Potentials for the Major Products of NO Electroreduction on Transition Metal Catalysts. Appl. Surf. Sci. 2021, 552, 149063 10.1016/j.apsusc.2021.149063.

[ref19] Katsounaros I.; Figueiredo M. C.; Chen X.; Calle-Vallejo F.; Koper M. T. M. Structure- and Coverage-Sensitive Mechanism of NO Reduction on Platinum Electrodes. ACS Catal. 2017, 7 (7), 4660–4667. 10.1021/acscatal.7b01069.

[ref20] Clayborne A.; Chun H.-J.; Rankin R. B.; Greeley J. Elucidation of Pathways for NO Electroreduction on Pt(111) from First Principles. Angew. Chem., Int. Ed. 2015, 54 (28), 8255–8258. 10.1002/anie.201502104. 26053610

[ref21] Wan H.; Bagger A.; Rossmeisl J. Electrochemical Nitric Oxide Reduction on Metal Surfaces. Angew. Chem. 2021, 133 (40), 22137–22143. 10.1002/ange.202108575. 34350689

[ref22] Chun H.-J.; Apaja V.; Clayborne A.; Honkala K.; Greeley J. Atomistic Insights into Nitrogen-Cycle Electrochemistry: A Combined DFT and Kinetic Monte Carlo Analysis of NO Electrochemical Reduction on Pt(100). ACS Catal. 2017, 7 (6), 3869–3882. 10.1021/acscatal.7b00547.

[ref23] Kresse G.; Furthmüller J. Efficient Iterative Schemes for Ab Initio Total-Energy Calculations Using a Plane-Wave Basis Set. Phys. Rev. B 1996, 54 (16), 11169–11186. 10.1103/PhysRevB.54.11169. 9984901

[ref24] Perdew J. P.; Burke K.; Ernzerhof M. Generalized Gradient Approximation Made Simple. Phys. Rev. Lett. 1996, 77 (18), 3865–3868. 10.1103/PhysRevLett.77.3865. 10062328

[ref25] Janthon P.; Kozlov S. M.; Viñes F.; Limtrakul J.; Illas F. Establishing the Accuracy of Broadly Used Density Functionals in Describing Bulk Properties of Transition Metals. J. Chem. Theory Comput. 2013, 9 (3), 1631–1640. 10.1021/ct3010326. 26587624

[ref26] Janthon P.; Luo S.; Kozlov S. M.; Vines F.; Limtrakul J.; Truhlar D. G.; Illas F. Bulk Properties of Transition Metals: A Challenge for the Design of Universal Density Functionals. J. Chem. Theory Comput. 2014, 10 (9), 3832–3839. 10.1021/ct500532v. 26588528

[ref27] Vega L.; Ruvireta J.; Viñes F.; Illas F. Jacob’s Ladder as Sketched by Escher: Assessing the Performance of Broadly Used Density Functionals on Transition Metal Surface Properties. J. Chem. Theory Comput. 2018, 14 (1), 395–403. 10.1021/acs.jctc.7b01047. 29182868

[ref28] Kresse G.; Joubert D. From Ultrasoft Pseudopotentials to the Projector Augmented-Wave Method. Phys. Rev. B 1999, 59 (3), 1758–1775. 10.1103/PhysRevB.59.1758.

[ref29] Methfessel M.; Paxton A. T. High-Precision Sampling for Brillouin-Zone Integration in Metals. Phys. Rev. B 1989, 40 (6), 3616–3621. 10.1103/PhysRevB.40.3616. 9992329

[ref30] Monkhorst H. J.; Pack J. D. Special Points for Brillouin-Zone Integrations. Phys. Rev. B 1976, 13 (12), 5188–5192. 10.1103/PhysRevB.13.5188.

[ref31] Lide D. R. CRC Handbook of Chemistry and Physics, 85th ed.; CRC Press, 2004.

[ref32] Granda-Marulanda L. P.; Rendón-Calle A.; Builes S.; Illas F.; Koper M. T. M.; Calle-Vallejo F. A Semiempirical Method to Detect and Correct DFT-Based Gas-Phase Errors and Its Application in Electrocatalysis. ACS Catal. 2020, 10 (12), 6900–6907. 10.1021/acscatal.0c01075.

[ref33] Urrego-Ortiz R.; Builes S.; Calle-Vallejo F. Fast Correction of Errors in the DFT-Calculated Energies of Gaseous Nitrogen-Containing Species. ChemCatChem. 2021, 13 (10), 2508–2516. 10.1002/cctc.202100125.

[ref34] Urrego-Ortiz R.; Builes S.; Calle-Vallejo F. Impact of Intrinsic Density Functional Theory Errors on the Predictive Power of Nitrogen Cycle Electrocatalysis Models. ACS Catal. 2022, 12 (8), 4784–4791. 10.1021/acscatal.1c05333. 35465243 PMC9017217

[ref35] Urrego-Ortiz R.; Builes S.; Calle-Vallejo F. Automated versus Chemically Intuitive Deconvolution of Density Functional Theory (DFT)-Based Gas-Phase Errors in Nitrogen Compounds. Ind. Eng. Chem. Res. 2022, 61 (36), 13375–13382. 10.1021/acs.iecr.2c02111. 36123997 PMC9479071

[ref36] Rendón-Calle A.; Builes S.; Calle-Vallejo F. Substantial Improvement of Electrocatalytic Predictions by Systematic Assessment of Solvent Effects on Adsorption Energies. Appl. Catal. B Environ. 2020, 276, 119147 10.1016/j.apcatb.2020.119147.

[ref37] Mathew K.; Sundararaman R.; Letchworth-Weaver K.; Arias T. A.; Hennig R. G. Implicit Solvation Model for Density-Functional Study of Nanocrystal Surfaces and Reaction Pathways. J. Chem. Phys. 2014, 140 (8), 084106 10.1063/1.4865107. 24588147

[ref38] Nørskov J. K.; Rossmeisl J.; Logadottir A.; Lindqvist L.; Kitchin J. R.; Bligaard T.; Jónsson H. Origin of the Overpotential for Oxygen Reduction at a Fuel-Cell Cathode. J. Phys. Chem. B 2004, 108 (46), 17886–17892. 10.1021/jp047349j. 39682080

[ref39] Abild-Pedersen F.; Greeley J.; Studt F.; Rossmeisl J.; Munter T. R.; Moses P. G.; Skúlason E.; Bligaard T.; Nørskov J. K. Scaling Properties of Adsorption Energies for Hydrogen-Containing Molecules on Transition-Metal Surfaces. Phys. Rev. Lett. 2007, 99 (1), 016105 10.1103/PhysRevLett.99.016105. 17678168

[ref40] Sakong S.; Groß A. The Importance of the Electrochemical Environment in the Electro-Oxidation of Methanol on Pt(111). ACS Catal. 2016, 6 (8), 5575–5586. 10.1021/acscatal.6b00931.

[ref41] Garcia-Ratés M.; García-Muelas R.; López N. Solvation Effects on Methanol Decomposition on Pd(111), Pt(111), and Ru(0001). J. Phys. Chem. C 2017, 121 (25), 13803–13809. 10.1021/acs.jpcc.7b05545.

[ref42] He Z.-D.; Hanselman S.; Chen Y.-X.; Koper M. T. M.; Calle-Vallejo F. Importance of Solvation for the Accurate Prediction of Oxygen Reduction Activities of Pt-Based Electrocatalysts. J. Phys. Chem. Lett. 2017, 8 (10), 2243–2246. 10.1021/acs.jpclett.7b01018. 28514862

[ref43] de Vooys A. C. A.; Koper M. T. M.; van Santen R. A.; van Veen J. A. R. Mechanistic Study of the Nitric Oxide Reduction on a Polycrystalline Platinum Electrode. Electrochim. Acta 2001, 46 (6), 923–930. 10.1016/S0013-4686(00)00678-2.

[ref44] Pérez-Gallent E.; Figueiredo M. C.; Katsounaros I.; Koper M. T. M. Electrocatalytic Reduction of Nitrate on Copper Single Crystals in Acidic and Alkaline Solutions. Electrochim. Acta 2017, 227, 77–84. 10.1016/j.electacta.2016.12.147.

[ref45] Wang Y.; Xu A.; Wang Z.; Huang L.; Li J.; Li F.; Wicks J.; Luo M.; Nam D.-H.; Tan C.-S.; Ding Y.; Wu J.; Lum Y.; Dinh C.-T.; Sinton D.; Zheng G.; Sargent E. H. Enhanced Nitrate-to-Ammonia Activity on Copper–Nickel Alloys via Tuning of Intermediate Adsorption. J. Am. Chem. Soc. 2020, 142 (12), 5702–5708. 10.1021/jacs.9b13347. 32118414

[ref46] Koper M. T. M. Analysis of Electrocatalytic Reaction Schemes: Distinction between Rate-Determining and Potential-Determining Steps. J. Solid State Electrochem 2013, 17 (2), 339–344. 10.1007/s10008-012-1918-x.

[ref47] Razzaq S.; Exner K. S. Materials Screening by the Descriptor Gmax(η): The Free-Energy Span Model in Electrocatalysis. ACS Catal. 2023, 13 (3), 1740–1758. 10.1021/acscatal.2c03997. 36776387 PMC9903997

[ref48] Peterson A. A.; Nørskov J. K. Activity Descriptors for CO2 Electroreduction to Methane on Transition-Metal Catalysts. J. Phys. Chem. Lett. 2012, 3 (2), 251–258. 10.1021/jz201461p.

[ref49] Koper M. T. M. Thermodynamic Theory of Multi-Electron Transfer Reactions: Implications for Electrocatalysis. J. Electroanal. Chem. 2011, 660 (2), 254–260. 10.1016/j.jelechem.2010.10.004.

[ref50] Man I. C.; Su H.-Y.; Calle-Vallejo F.; Hansen H. A.; Martínez J. I.; Inoglu N. G.; Kitchin J.; Jaramillo T. F.; Nørskov J. K.; Rossmeisl J. Universality in Oxygen Evolution Electrocatalysis on Oxide Surfaces. ChemCatChem. 2011, 3 (7), 1159–1165. 10.1002/cctc.201000397.

