# Supplementary material for: Extracting Features of Active Transition Metal Electrodes for NO Electroreduction with Catalytic Matrices
Source: ACS Appl Mater Interfaces. 2023 Apr 25;15(18):22176–83. doi: 10.1021/acsami.3c03385 (PMC10176317; doi:10.1021/acsami.3c03385)
Supplement: Supplementary file 1 — am3c03385_si_001.pdf [file am3c03385_si_001.pdf]

Supporting Information

Extracting features of active transition metal electrodes for NO electroreduction with catalytic matrices

Eleonora Romeo,[1] María Fernanda Lezana-Muralles,[1] Francesc Illas[1,*], and Federico Calle-Vallejo[2,3,*]

[1]Department de Ciència de Materials i Química Física & Institut de Química Teòrica i Computacional (IQTCUB), Universitat de Barcelona, C/ Martí i Franquès 1, 08028 Barcelona, Spain.

[2] Nano-Bio Spectroscopy Group and European Theoretical Spectroscopy Facility (ETSF), Department of Polymers and Advanced Materials: Physics, Chemistry and Technology, University of the Basque Country UPV/EHU, Av. Tolosa 72, 20018 San Sebastián, Spain.

[3] IKERBASQUE, Basque Foundation for Science, Plaza de Euskadi 5, 48009 Bilbao, Spain.

*Corresponding authors: francesc.illas@ub.edu ; federico.calle@ehu.es

Index of Contents

S1. Additional computational details S2

S2. Micro-solvation method S6

S3. Activity assessment S7

S4. Selectivity matrices S8

S6. Free energies of adsorption in vacuum S12

S7. Solvation contributions to the free energies of adsorption S13

S8. Adsorption-energy scaling relations S14

S9. Slopes of the scaling relations S20

S10. Multivariate regressions S20

S11. References S23

S1. Additional computational details

All surfaces were represented by periodic slabs consisting of four atomic layers (plus the metal adatoms for 4AD@(100) and 3AD@(111)), except for (100) facets which require five layers to achieve converged adsorption energies.[1] The slabs were modelled with the converged PBE lattice constants reported in the Supporting Information, Table S1. For the (111) and (100) surfaces, (2×2) supercells were used containing 4 atoms per atomic layer. For the (211) surface, (2×1) supercells containing 6 atoms per layer were used. For the (211)k surface, a (3×1) supercell was built with 9 atoms per layer except for the topmost atomic layer, which contained 8 atoms. The model for the 4AD@(100) is a (3×3) (100) supercell with 9 atoms per layer and an additional square of 4 atoms on top. The model for the 3AD@(111) is a (3×3) (111) supercell with a with 9 atoms per layer and an additional triangle of 3 atoms on top. Additional details on the slabs and their monodentate and bidentate adsorption sites are provided in Tables S1-S2 and Figures S1-S2. Co was assumed to be an fcc metal instead of an hcp metal to simplify the analysis in terms of coordination numbers, since the other eight transition metals in this study crystallize in the fcc system. Based on previous works,[2] we do not expect significant changes as a result of that approximation. The numerical integration in the reciprocal space was carried out using Monkhorst-Pack[3] special k-points grids of 6×6×1 for the (100) and (111) facets, 6×4×1 for (211) slabs, 4×4×1 for (211)k, and 4×4×1 for 4AD@Cu(111) and 3AD@(111) model surfaces, which guaranteed convergence of the adsorption energies within ±0.05 eV. In all geometry optimizations, the adsorbates, the two uppermost layers (and the metal adatoms, whenever present) were allowed to relax in all directions, while the remaining metal atoms were fixed at the optimized bulk coordinates. On average, 6 different initial configurations were relaxed for each adsorbate on each active site, including, when possible, monodentate and bidentate configurations. For all adsorbates, energy minimum configurations were confirmed as stationary points by means of appropriate vibrational analysis with all frequencies computed in the harmonic approximation and as finite differences of analytic gradients. Vibrational frequencies for the gas-phase species were also computed to be able to estimate the zero-point energies (ZPEs).

Nitric oxide and hydrogen were used to assess the adsorption energies of *NO, *NHO and *NOH. The gas-phase correction for NO(g) is semiempirically obtained from $\frac{1}{2}N_2 + \frac{1}{2}O_2 \rightarrow NO$, which implies that the corrections for N_2 and O_2 need to be known beforehand. The one for N_2 is calculated from $2NH_3 \rightarrow N_2 + 3H_2$ and is 0.33 eV for PBE. In turn, the

correction for O_2 is calculated from $2H_2O \rightarrow O_2 + 2H_2$ and is -0.45 eV for PBE. With these two corrections we find that NO(g) is relatively well described by PBE, as the error in its formation energy with respect to experiments is as low as -0.07 eV. Hence, the total energy of NO(g) used to evaluate the adsorption energies was shifted by this much to correct its gas-phase error.

The contributions of solvent-adsorbate interactions to the free energy were evaluated using different solvation approaches. First, we used an implicit solvation method (VASPsol[4,5]) incorporated into VASP, which simulates the solvent as a continuum with a given dielectric constant and, hence, includes only electrostatic effects. Second, we used an ad hoc approach based on the constant values calculated by Clayborne et al for *NO, *NOH and *NHO on Pt(111).[6] The third approach is a micro-solvation method considering just the water molecules interacting directly with the adsorbates through hydrogen bonds, such that the stabilization granted by the solvent arises to a great extent from the first solvation shell.[7] More details about this method can be found in section S2. The fourth approach takes the largest (most negative) contribution among implicit and micro-solvation. We refer to it as the mixed solvation approach. Because of the large computational burden, micro-solvation corrections were explicitly computed for the (111) and (100) slabs only. The values found for the adsorbates on the (111) surfaces were used for the facets with hexagonal symmetry, namely, (211), (211)k, and 3AD@(111) slabs, whereas those for (100) terraces were extrapolated for adsorbates at facets with square symmetry, namely, 4AD@(100) slabs.

Table S1. Calculated lattice constant of the metals under study.

Metal	Lattice constant (Å)
Co	3.52
Ni	3.52
Cu	3.64
Rh	3.85
Pd	3.96
Ag	4.17
Ir	3.88
Pt	3.98
Au	4.18

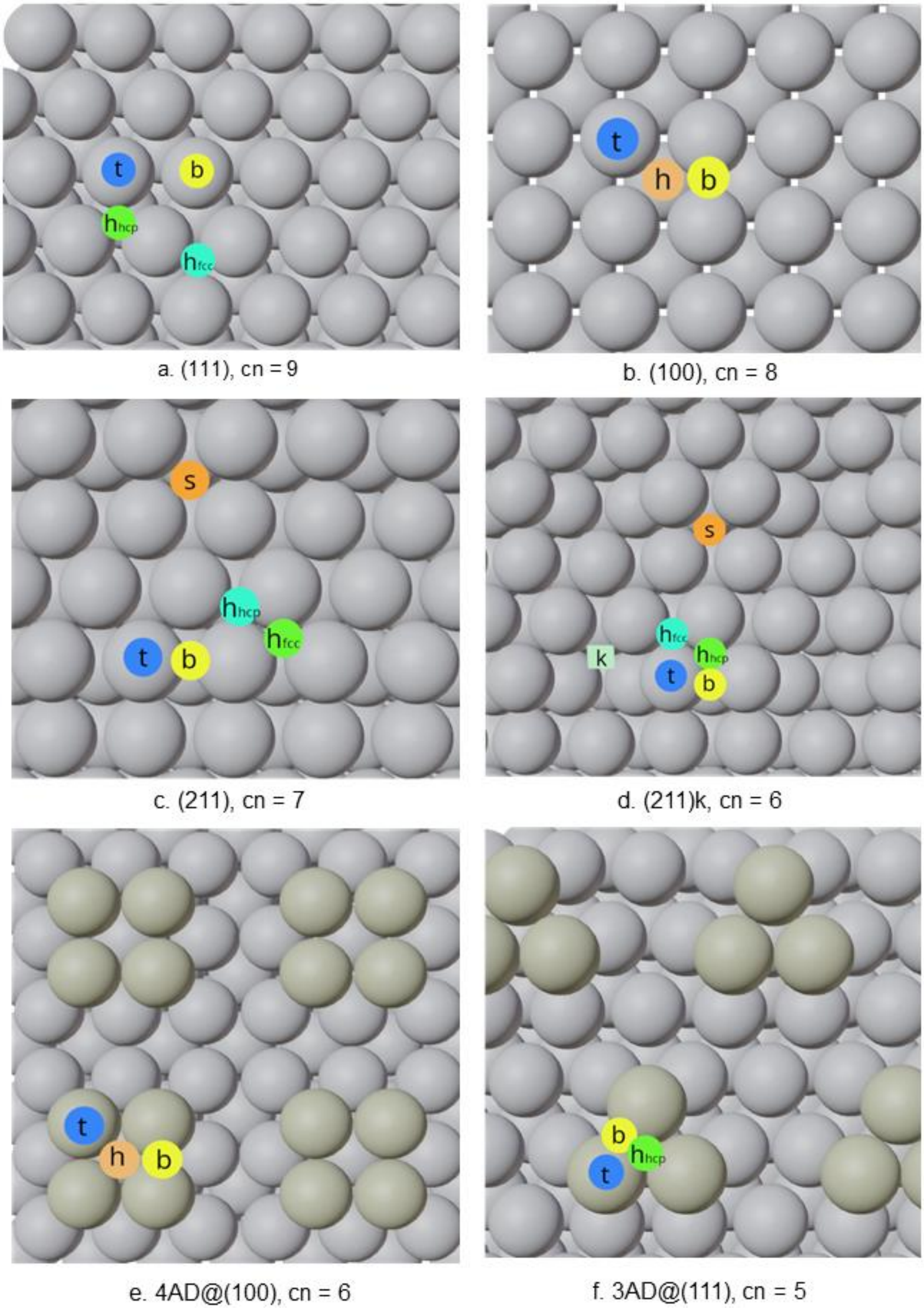

Figure S1. Structures of the monodentate adsorption sites under study. The monodentate adsorption sites are marked as follows: fcc threefold hollow site (h_{fcc}), hcp threefold hollow site (h_{hcp}), fourfold hollow sites (h), atop site (top), bridge site (b), square site the (211) surface (s). Surface atoms are shown in grey, metal adatoms in ochre.

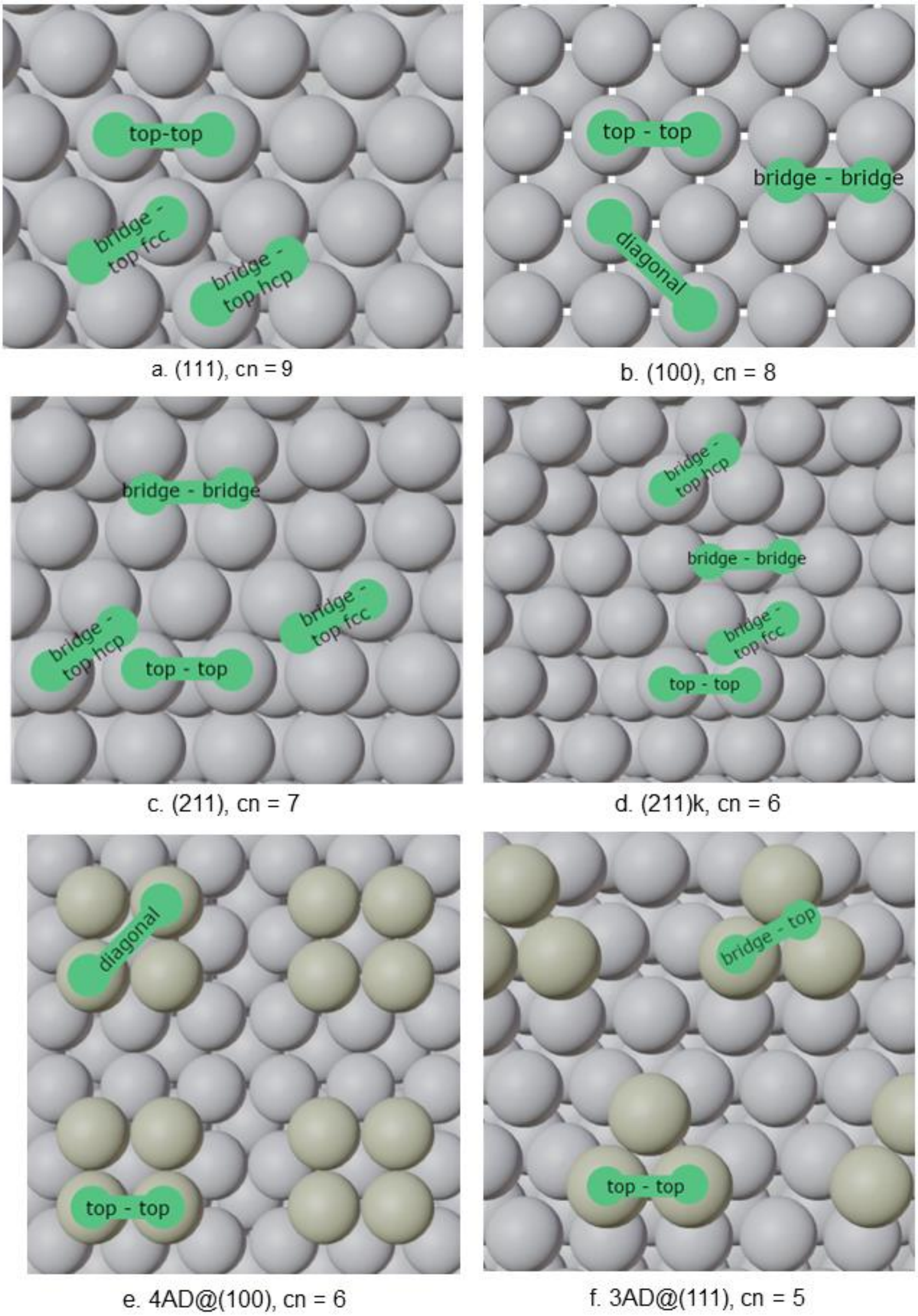

Figure S2. Structures of the bidentate adsorption sites under study. Surface atoms are shown in grey, metal adatoms in ochre.

S2. Micro-solvation method

In this section we briefly revisit the micro-solvation method by Rendón-Calle et al.[7] If water is the solvent, explicit water molecules are subsequently added around the adsorbate until its first solvation shell is complete. In other words, solvation energies are assessed by consecutively adding water molecules in the vicinity of the adsorbate in configurations that allow hydrogen bond formation. After adding each water molecule, it is necessary to compare the stabilization provided by water to the adsorbate with the water self-solvation energy on the specific material and facet to decide whether the new water molecule is truly part of the first solvation shell of the adsorbate. We estimated the water self-solvation criteria for the (111) and (100) surfaces of Co, Ni, Cu, Rh, Pd, Ag, Ir, Pt and Au considering a cluster of four water molecules interacting with the surface, which are simultaneously the adsorbate (at the center) and the solvent (in the periphery), see Figure S3. To avoid lateral interactions between water molecules, we used 4×4 (111) and 4×4 (100) slabs.

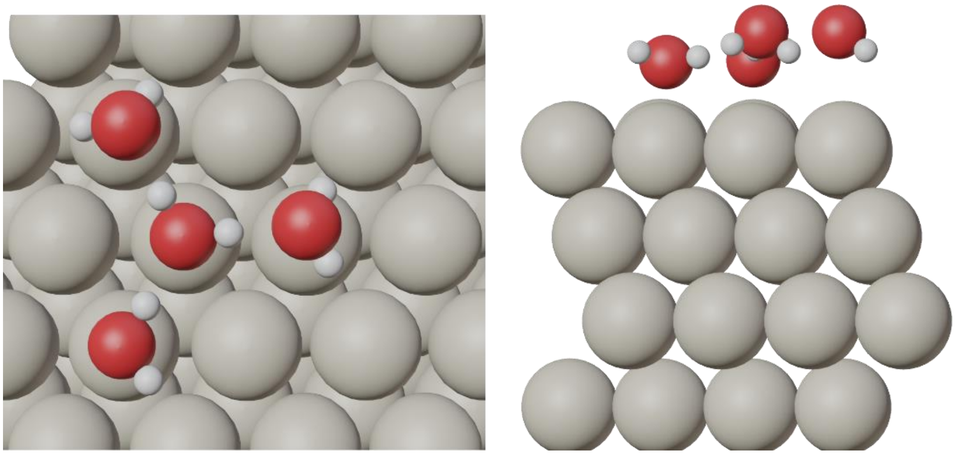

Figure S3. Cluster of four water molecule, top (left) side (right) view. The central water molecule is the adsorbate while the surrounding three water molecules are the first solvation shell.

The energy of the solvated water molecule at the center is evaluated both in the solvated environment and in vacuum. The energies of those systems are:

$$\Delta E^{solv}_{*H_2O} = E_{*H_2O+3*H_2O} - 4E_{H_2O} - E_* \quad \text{(S1)}$$

$$\Delta E^{vac}_{*H_2O} = E_{*H_2O} - E_{H_2O} - E_* \quad \text{(S2)}$$

The total self-solvation of water is the difference between Equations S1 and S2:

$$\Omega^{tot}_{H_2O} = \Delta E^{solv}_{*H_2O} - 4\Delta E^{vac}_{*H_2O} \quad \text{(S3)}$$

Dividing Equation S3 by three, which is the number of solvating water molecules (and of hydrogen bonds), we obtain the self-solvation correction per hydrogen bond:

$$\Omega_{\mathrm{H}_2O} = \frac{\Omega^{tot}_{H_2O}}{3} = \frac{1}{3}\Delta E^{solv}_{*H_2O} - \frac{4}{3}\Delta E^{vac}_{*H_2O} \quad \text{(S4)}$$

where Ω_{H_2O} is the water self-solvation energy used as a decision criterion to evaluate the number of water molecules solvating *NO, *NOH and *NHO, respectively. For clarity, in the following we explain the procedure for *NOH. The first solvation process is represented by:

$$* NOH + [* H_2O + *] \rightarrow [* NOH + * H_2O] + * \quad \text{(S5)}$$

where the brackets represent solvated states (e.g., $[* NOH + * H_2O]$ represents *NOH solvated by a water molecule). The gain in energy by hydrogen bonding is to be evaluated. For one water molecule, the solvation energy is described by:

$$\Delta E^{1H_2O}_{solv} = E_{[*NOH+*H_2O]} + E_* - E_{*NOH} - E_{[*H_2O+*]} \quad \text{(S6)}$$

and the gain is written as:

$$g^{1H_2O} = (E_{[*NOH+*H_2O]} + 2\Omega_{H_2O}) + E_* - E_{*NOH} - (E_{[1*H_2O]} + 3\Omega_{H_2O}) \quad \text{(S7)}$$

Equation S7 takes into account the hydrogen bonds of the solvating water molecule with the water cluster without and with the interaction with the adsorbate. If $g^{1H_2O} \leq 0$, the added water molecule stabilizes the system, and the iterative process continues. For example, for Cu(111) we calculated $\Omega_{H_2O} = -0.24\, eV$, so one gets $\Delta E^{1H_2O}_{solv} = -0.29\, eV$, and $g^{1H_2O} = -0.05\, eV < 0$. Hence, we can continue the test with a second water molecule. The solvation of *NOH by two H_2O is:

$$* NOH + 2[* H_2O + *] \rightarrow [* NOH + 2 * H_2O] + 2 * \quad \text{(S8)}$$

The resulting solvation energy is:

$$\Delta E^{2H_2O}_{solv} = E_{[*NOH+2*H_2O]} + 2\, E_* - E_{*NOH} - E_{2[*H_2O+*]} \quad \text{(S9)}$$

The gain for adding a second water molecule is evaluated with the equation:

$$g^{2H_2O} = (E_{[*NOH+2*H_2O]} + 4\Omega_{H_2O}) + 2E_* - E_{*NOH} - 2(E_{[1*H_2O]} + 3\Omega_{H_2O}) - \Delta E^{1H_2O}_{solv} \quad \text{(S10)}$$

For Cu(111) we obtain $\Delta E^{2H_2O}_{solv} = -0.30\, eV$ and $g^{1H_2O} = 0.17\, eV > 0$, so we stop the process and consider n = 1 and $\Delta E_{solv} = -0.29\, eV$ as the solvation correction for *NOH.

S3. Activity assessment

For NO electroreduction, we approximate the onset potential using Equation S11 (Equation 3 in the main text), which is the potential required for *NO hydrogenation to either *NOH or *NHO. This assumes that *NO hydrogenation is the potential-limiting step of NO electroreduction or requires a potential close to it. The computed values for the onset potential are reported in Table S9.

$$U_{onset} \approx \frac{\min(\Delta G_{*NOH}, \Delta G_{*NHO}) - \Delta G_{*NO}}{-1e^-} \quad \text{(S11)}$$

S4. Selectivity matrices

The most stable products of *NO hydrogenation for all the surfaces in vacuum and with solvation corrections are shown below.

Table S2. Most stable *NO hydrogenation product in vacuum.

**Surface** *cn*	**(111)** 9	**(100)** 8	**(211)** 7	**(211)k** 6	**4AD@(100)** 6	**3AD@(111)** 5
**Co**	*NOH	*NHO	both	*NHO	*NHO	*NHO
**Rh**	both	*NHO	both	*NHO	*NHO	*NHO
**Ir**	both	*NHO	*NHO	*NHO	*NHO	*NHO
**Ni**	*NOH	*NHO	both	both	*NHO	*NHO
**Pd**	*NOH	*NHO	*NHO	both	*NHO	both
**Pt**	*NOH	*NHO	*NHO	*NHO	*NHO	*NHO
**Cu**	both	*NHO	*NHO	*NHO	*NHO	*NHO
**Ag**	*NHO	*NHO	*NHO	*NHO	*NHO	*NHO
**Au**	*NHO	*NHO	*NHO	*NHO	*NHO	*NHO

Table S3. Most stable *NO hydrogenation product with implicit[4,5] solvation corrections.

**Surface** *cn*	**(111)** 9	**(100)** 8	**(211)** 7	**(211)k** 6	**4AD@(100)** 6	**3AD@(111)** 5
**Co**	*NOH	*NHO	*NOH	*NHO	*NHO	*NHO
**Rh**	both	*NHO	both	*NHO	*NHO	*NHO
**Ir**	both	*NHO	*NHO	*NHO	*NHO	*NHO
**Ni**	*NOH	*NHO	*NOH	both	*NHO	*NHO
**Pd**	*NOH	*NHO	*NHO	both	*NHO	both
**Pt**	*NOH	*NHO	*NHO	both	*NHO	both
**Cu**	both	*NHO	both	*NHO	*NHO	*NHO
**Ag**	*NHO	*NHO	*NHO	*NHO	*NHO	*NHO
**Au**	*NHO	*NHO	*NHO	*NHO	*NHO	*NHO

Table S4. Most stable *NO hydrogenation product with ad-hoc solvation corrections ($\Delta E_{solv(*NOH)}$ = -0.31 eV, $\Delta E_{solv(*NHO)}$ = -0.23 eV.[6]

**Surface** *cn*	**(111)** 9	**(100)** 8	**(211)** 7	**(211)k** 6	**4AD@(100)** 6	**3AD@(111)** 5
**Co**	*NOH	*NHO	*NOH	*NHO	*NHO	*NHO
**Rh**	*NOH	*NHO	both	*NHO	*NHO	*NHO
**Ir**	*NOH	*NHO	*NHO	*NHO	*NHO	*NHO
**Ni**	*NOH	both	*NOH	both	*NHO	*NHO
**Pd**	*NOH	both	both	*NOH	*NHO	both
**Pt**	*NOH	*NHO	*NHO	*NHO	*NHO	both
**Cu**	both	*NHO	both	*NHO	*NHO	*NHO
**Ag**	*NHO	*NHO	*NHO	*NHO	*NHO	*NHO
**Au**	*NHO	*NHO	*NHO	*NHO	*NHO	*NHO

Table S5. Most stable *NO hydrogenation product with micro-solvation corrections.[7]

**Surface** *cn*	**(111)** 9	**(100)** 8	**(211)** 7	**(211)k** 6	**4AD@(100)** 6	**3AD@(111)** 5
**Co**	*NOH	*NHO	both	*NHO	*NHO	*NHO
**Rh**	both	*NHO	both	*NHO	*NHO	*NHO
**Ir**	both	*NHO	*NHO	*NHO	*NHO	*NHO
**Ni**	*NOH	*NHO	*NOH	both	*NHO	*NHO
**Pd**	*NOH	*NOH	*NOH	*NOH	both	*NOH
**Pt**	*NOH	both	*NHO	both	*NOH	both
**Cu**	both	*NHO	*NHO	*NHO	*NHO	*NHO
**Ag**	*NHO	*NHO	*NHO	*NHO	*NHO	*NHO
**Au**	*NHO	*NHO	*NHO	*NHO	*NHO	*NHO

Table S6. Most stable adsorbate in vacuum and with different solvation models for Co, Rh, and Ir. The averages do not include the data in vacuum.

solvation model	**%**			
	***NOH**	***NHO**	**both**	
in vacuum	6%	72%	17%	6%
implicit	11%	72%	17%	0%
ad hoc	22%	72%	6%	0%
micro-solvation	6%	72%	17%	6%
mixed	6%	72%	17%	6%
average	(11 ± 8)%	(72 ± 0)%	(14 ± 6)%	(3 ± 3)%

Table S7. Most stable adsorbate in vacuum and with different solvation models for Ni, Pd, and Pt. The averages do not include the data in vacuum.

solvation model	**%**			
	***NOH**	***NHO**	**both**	
in vacuum	17%	61%	11%	11%
implicit	22%	50%	17%	11%
ad hoc	28%	39%	11%	22%
micro-solvation	50%	22%	22%	6%
mixed	33%	22%	28%	17%
average	(33 ± 12)%	(33 ± 14)%	(20 ± 7)%	(14 ± 7)%

Table S8. Most stable adsorbate in vacuum and with different solvation models for Cu, Ag, and Au. The averages do not include the data in vacuum.

solvation model	%			
	***NOH**	***NHO**	**both**	
in vacuum	0%	94%	6%	0%
implicit	0%	89%	5%	6%
ad hoc	0%	89%	5%	6%
micro-solvation	0%	94%	0%	6%
mixed	0%	94%	0%	6%
average	0%	(92 ± 3)%	(3 ± 3)%	6%

S5. Most stable adsorption configurations

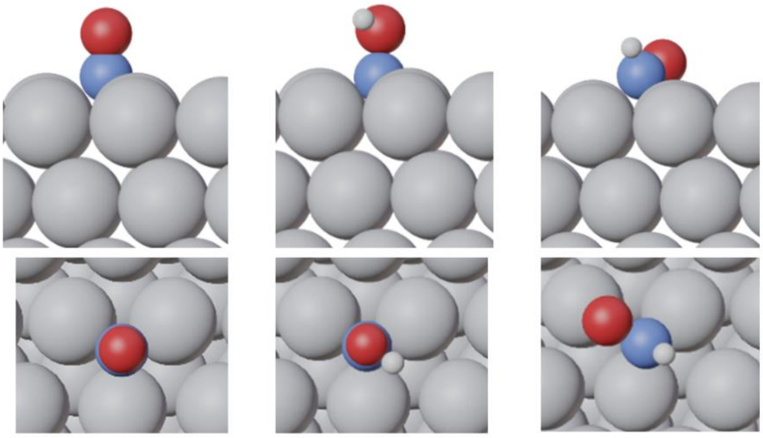

a) *NO b) *NOH c) *NHO

Figure S4. Side and top views for the most stable configurations of *NO, *NOH and *NHO on Pt(111).

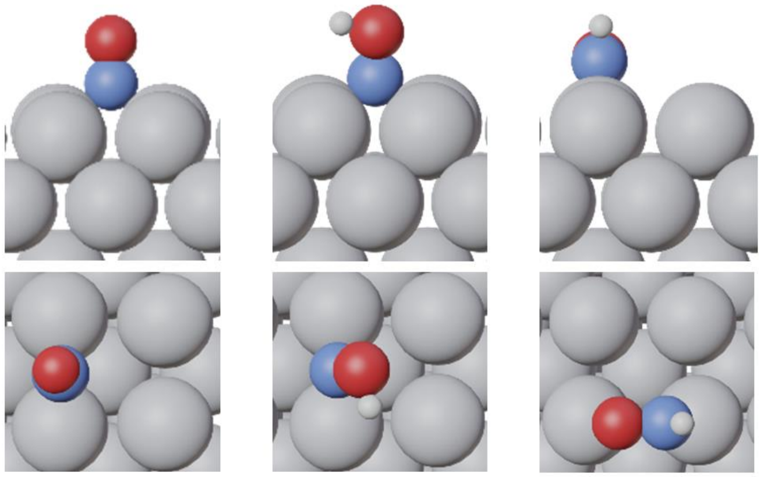

a) *NO b) *NOH c) *NHO

Figure S5. Side and top views for the most stable configurations of *NO, *NOH and *NHO on Pt(100).

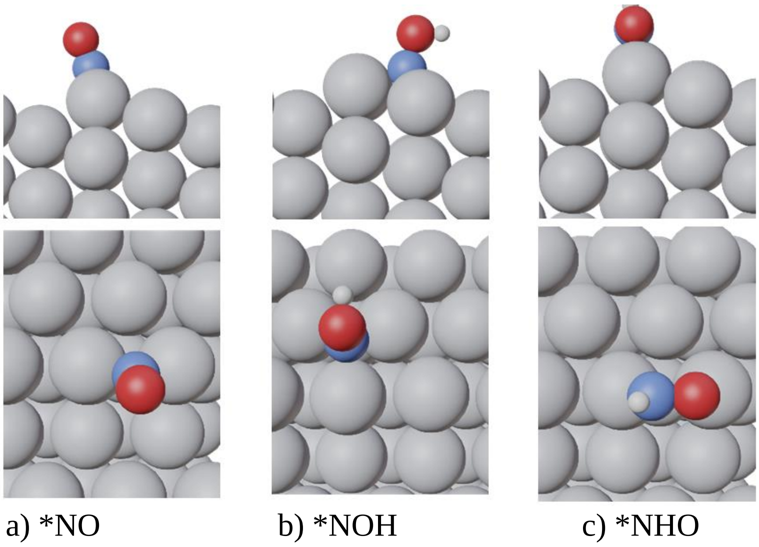

Figure S6. Side and top views for the most stable configurations of *NO, *NOH and *NHO on Pt(211).

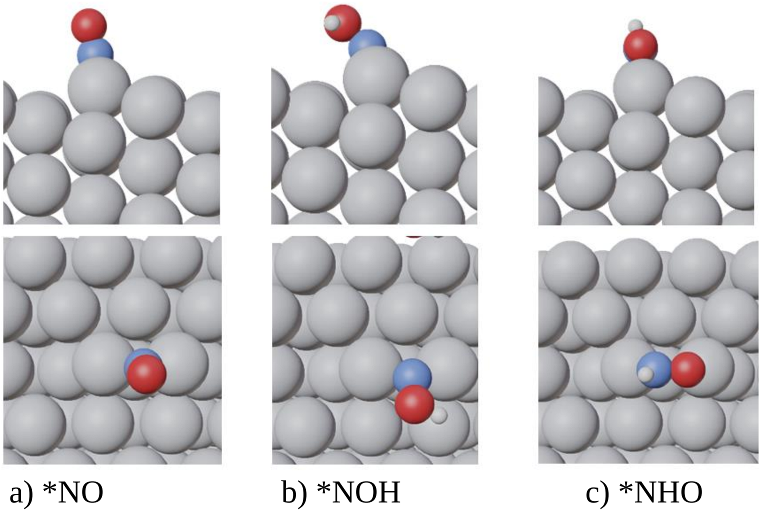

Figure S7. Side and top views for the most stable configurations of *NO, *NOH and *NHO on Pt(211)k.

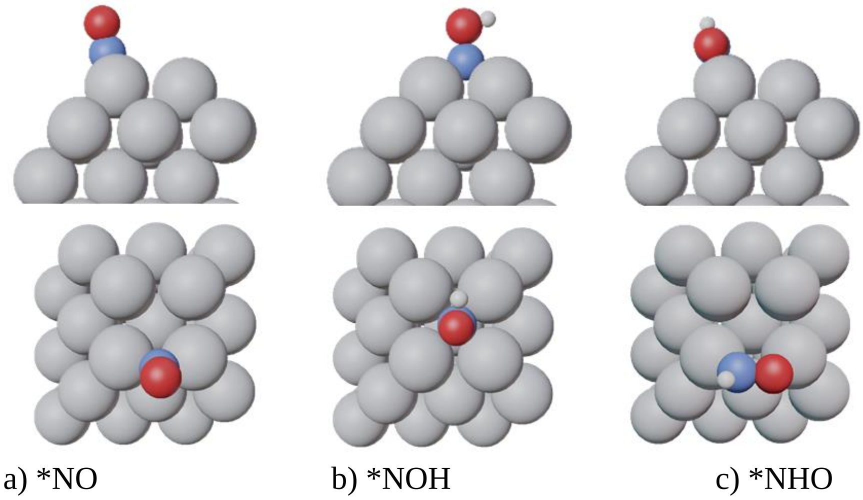

Figure S8. Side and top views for the most stable configurations of *NO, *NOH and *NHO on 4AD@Pt(100).

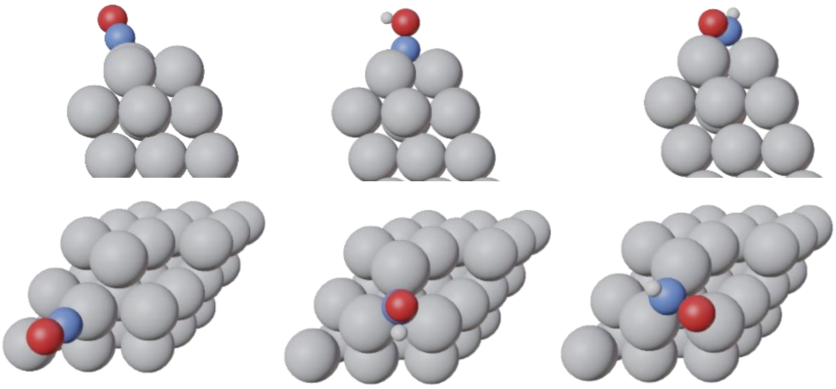

Figure labels: a) *NO b) *NOH c) *NHO

Figure S9. Side and top views for the most stable configurations of *NO, *NOH and *NHO on 3AD@Pt(111).

S6. Free energies of adsorption in vacuum

Table S9. Adsorption energies of *NO, *NOH and *NHO for all considered metals and surfaces in vacuum. The onset potential in the rightmost column is calculated using Equation S11, which uses the adsorption energies in this table but corrected with the solvation energies corresponding to the mixed scheme in Table S10.

Surface	Metal	$\Delta G_{NO,vac}$ (eV)	$\Delta G_{NOH,vac}$ (eV)	$\Delta G_{NHO,vac}$ (eV)	U_{onset} (V)
111	Co	-1.91	-1.59	-1.25	-0.28
	Ni	-1.91	-1.47	-1.22	-0.33
	Cu	-0.69	-0.39	-0.38	-0.14
	Rh	-1.84	-1.42	-1.37	-0.33
	Pd	-1.71	-1.02	-0.89	-0.33
	Ag	0.09	0.72	0.27	0.11
	Ir	-1.28	-1.18	-1.11	-0.02
	Pt	-1.32	-1.00	-0.80	0.07
	Au	0.25	0.84	0.33	0.14
100	Co	-1.98	-1.91	-2.21	0.28
	Ni	-1.99	-1.91	-2.04	0.18
	Cu	-0.65	-0.74	-0.97	0.47
	Rh	-2.00	-1.70	-1.98	0.10
	Pd	-1.61	-1.11	-1.26	-0.21
	Ag	-0.01	0.42	0.08	0.05
	Ir	-1.86	-1.21	-1.93	0.07
	Pt	-1.72	-1.02	-1.40	-0.18
	Au	0.07	0.77	0.18	-0.02
211	Co	-2.06	-1.67	-1.63	-0.34
	Ni	-2.04	-1.55	-1.47	-0.38
	Cu	-0.75	-0.44	-0.62	0.04
	Rh	-2.17	-1.58	-1.65	-0.43
	Pd	-1.75	-0.98	-1.10	-0.41
	Ag	-0.02	0.67	0.00	0.28

	Ir	-2.23	-1.84	-2.08	-0.06
	Pt	-1.82	-0.90	-1.29	-0.33
	Au	-0.16	0.55	0.08	-0.01
211k	Co	-1.98	-1.51	-1.72	-0.22
	Ni	-2.06	-1.56	-1.59	-0.38
	Cu	-0.77	-0.50	-0.82	0.22
	Rh	-2.22	-1.49	-1.69	-0.43
	Pd	-1.77	-1.06	-1.02	-0.36
	Ag	0.01	0.58	-0.08	0.38
	Ir	-2.61	-1.53	-2.19	-0.33
	Pt	-1.51	-1.13	-1.31	0.02
	Au	-0.18	0.64	0.00	0.04
4AD @ 100	Co	-1.99	-1.72	-2.40	0.46
	Ni	-2.02	-1.69	-2.04	0.00
	Cu	-0.79	-0.77	-1.15	0.45
	Rh	-2.29	-1.60	-2.11	-0.10
	Pd	-1.70	-0.97	-1.17	-0.39
	Ag	-0.08	0.46	-0.05	0.03
	Ir	-2.66	-1.74	-2.29	-0.33
	Pt	-1.63	-1.05	-1.29	-0.13
	Au	-0.23	0.60	0.00	-0.09
3AD @ 111	Co	-2.13	-1.79	-2.05	-0.04
	Ni	-2.36	-1.85	-2.03	-0.25
	Cu	-0.93	-0.78	-1.05	0.30
	Rh	-2.28	-1.79	-2.01	-0.18
	Pd	-1.97	-1.12	-1.15	-0.50
	Ag	0.02	0.51	-0.05	0.36
	Ir	-2.92	-1.93	-2.41	-0.42
	Pt	-2.14	-1.46	-1.58	-0.28
	Au	-0.34	0.39	-0.21	0.10

S7. Solvation contributions to the free energies of adsorption

Table S10. Solvation contributions to the free energies of adsorption using implicit[4,5] and micro-solvation[7] methods for *NO, *NOH and *NHO for all the metals and surfaces. The micro-solvation corrections is computed for (111) and (100) surfaces and applied respectively to (211), (211)k, 3AD@(111), and 4AD@(100).

Surface	Metal	Implicit solvation			Micro-solvation			Mixed solvation		
		ΔE_{solv} NO	ΔE_{solv} NOH	ΔE_{solv} NHO	ΔE_{solv} NO	ΔE_{solv} NOH	ΔE_{solv} NHO	ΔE_{solv} NO	ΔE_{solv} NOH	ΔE_{solv} NHO
111	Co	-0.04	-0.15	-0.11	-0.22	-0.26	-0.25	-0.22	-0.26	-0.25
	Ni	-0.03	-0.17	-0.12	-0.15	-0.26	-0.23	-0.15	-0.26	-0.23
	Cu	-0.04	-0.17	-0.12	-0.21	-0.29	-0.38	-0.21	-0.29	-0.38
	Rh	-0.02	-0.18	-0.13	-0.15	-0.24	-0.24	-0.15	-0.24	-0.24
	Pd	-0.02	-0.19	-0.14	0.00	-0.37	0.00	-0.02	-0.37	-0.14
	Ag	-0.02	-0.19	-0.12	0.00	-0.29	-0.31	-0.02	-0.29	-0.31
	Ir	-0.02	-0.16	-0.14	-0.16	-0.25	-0.25	-0.16	-0.25	-0.25
	Pt	-0.02	-0.19	-0.15	0.00	-0.41	-0.19	-0.02	-0.41	-0.19

	Au	-0.01	-0.19	-0.12	0.00	-0.36	-0.23	-0.01	-0.36	-0.23
100	Co	0.00	-0.11	-0.11	-0.10	0.00	-0.16	-0.10	-0.11	-0.16
	Ni	0.01	-0.12	-0.12	0.00	0.00	0.00	0.00	-0.12	-0.12
	Cu	-0.04	-0.13	-0.16	0.00	-0.17	-0.18	-0.04	-0.17	-0.18
	Rh	-0.01	-0.09	-0.13	0.00	-0.16	0.00	-0.01	-0.16	-0.13
	Pd	0.04	-0.10	-0.12	0.00	-0.30	0.00	0.00	-0.30	-0.12
	Ag	0.00	-0.17	-0.14	0.00	-0.24	0.00	0.00	-0.24	-0.14
	Ir	-0.03	-0.30	-0.13	-0.12	-0.23	0.00	-0.12	-0.30	-0.13
	Pt	0.00	0.03	-0.14	0.00	-0.45	0.00	0.00	-0.45	-0.14
	Au	-0.03	-0.14	-0.13	0.00	-0.38	0.00	-0.03	-0.38	-0.13
211	Co	0.02	-0.19	-0.05	-0.22	-0.26	-0.25	-0.22	-0.26	-0.25
	Ni	-0.02	-0.21	-0.12	-0.15	-0.26	-0.23	-0.15	-0.26	-0.23
	Cu	-0.03	-0.19	-0.06	-0.21	-0.29	-0.38	-0.21	-0.29	-0.38
	Rh	0.01	-0.22	-0.13	-0.15	-0.24	-0.24	-0.15	-0.24	-0.24
	Pd	-0.01	-0.17	-0.19	0.00	-0.37	0.00	-0.01	-0.37	-0.19
	Ag	0.01	-0.14	-0.09	0.00	-0.29	-0.31	0.00	-0.29	-0.31
	Ir	0.00	-0.22	-0.15	-0.16	-0.25	-0.25	-0.16	-0.25	-0.25
	Pt	0.00	-0.20	-0.190	0.00	-0.41	-0.195	0.00	-0.41	-0.19
	Au	0.00	-0.23	-0.15	0.00	-0.36	-0.23	0.00	-0.36	-0.23
211k	Co	0.03	-0.16	-0.08	-0.22	-0.26	-0.25	-0.22	-0.26	-0.25
	Ni	-0.03	-0.14	-0.11	-0.15	-0.26	-0.23	-0.15	-0.26	-0.23
	Cu	-0.04	-0.16	-0.09	-0.21	-0.29	-0.38	-0.21	-0.29	-0.38
	Rh	0.01	-0.21	-0.11	-0.15	-0.24	-0.24	-0.15	-0.24	-0.24
	Pd	-0.02	-0.18	-0.12	0.00	-0.37	0.00	-0.02	-0.37	-0.12
	Ag	-0.02	-0.14	-0.08	0.00	-0.29	-0.31	-0.02	-0.29	-0.31
	Ir	0.01	-0.24	-0.15	-0.16	-0.25	-0.25	-0.16	-0.25	-0.25
	Pt	0.00	-0.29	-0.20	0.00	-0.41	-0.19	0.00	-0.41	-0.20
	Au	-0.01	-0.19	-0.14	0.00	-0.36	-0.23	-0.01	-0.36	-0.23
4AD@100	Co	-0.08	-0.17	-0.05	-0.10	0.00	-0.16	-0.10	-0.17	-0.16
	Ni	-0.03	-0.17	0.03	0.00	0.00	0.00	-0.03	-0.17	0.00
	Cu	-0.09	-0.18	-0.07	0.00	-0.17	-0.18	-0.09	-0.18	-0.18
	Rh	-0.02	-0.18	-0.10	0.00	-0.16	0.00	-0.02	-0.18	-0.10
	Pd	0.00	-0.21	-0.14	0.00	-0.30	0.00	0.00	-0.30	-0.14
	Ag	-0.04	-0.18	-0.10	0.00	-0.24	0.00	-0.04	-0.24	-0.10
	Ir	0.00	-0.21	-0.16	-0.12	-0.23	0.00	-0.12	-0.23	-0.16
	Pt	-0.01	-0.27	-0.18	0.00	-0.45	0.00	-0.01	-0.45	-0.18
	Au	0.00	-0.23	-0.15	0.00	-0.38	0.00	0.00	-0.38	-0.15
3AD @ 111	Co	-0.20	-0.23	-0.16	-0.22	-0.26	-0.25	-0.22	-0.26	-0.25
	Ni	-0.06	-0.16	-0.08	-0.15	-0.26	-0.23	-0.15	-0.26	-0.23
	Cu	-0.05	-0.16	-0.09	-0.21	-0.29	-0.38	-0.21	-0.29	-0.38
	Rh	-0.05	-0.16	-0.09	-0.15	-0.24	-0.24	-0.15	-0.24	-0.24
	Pd	-0.02	-0.21	-0.15	0.00	-0.37	0.00	-0.02	-0.37	-0.15
	Ag	-0.01	-0.16	-0.09	0.00	-0.29	-0.31	-0.01	-0.29	-0.31
	Ir	-0.03	-0.19	-0.12	-0.16	-0.25	-0.25	-0.16	-0.25	-0.25
	Pt	0.00	-0.24	-0.18	0.00	-0.41	-0.19	0.00	-0.41	-0.19
	Au	0.00	-0.22	-0.16	0.00	-0.36	-0.23	0.00	-0.36	-0.23

S8. Adsorption-energy scaling relations

In Figure S10, we plot ΔG_{NOH} and ΔG_{NHO} for the nine metals and six facets as a function of ΔG_{NO}. All values in the figure are reported in Tables S9-S10. In Figure S10 ΔG_{NHO} vs ΔG_{NO} can be described by a single line, despite the differences in coordination number of the adsorption sites, and the same can be said of *NOH vs *NO. This is because scaling relations

display structure-insensitive offsets when their slopes are close to 1,[8,9] or more generally, when the two adsorbates bind similarly to the surface.[10–13]

As solvent-adsorbate effects are generally stabilizing, the corresponding scaling lines have more negative offsets and are, thus, below the line of the calculations in vacuum. Moreover, implicit solvation can significantly modify the slopes of the scaling relations among *O-bound adsorbates.[14] Hence, to rationalize the influence of solvation on the slopes of the scaling relations, Figure S11plots them for each surface in vacuum and with the different solvation models (see also Table S11). According to Figure S11, the coordination of the adsorption site does not play a significant role on the slope and a single line assembling all metals and surface sites is enough to capture the trends. Nonetheless, Figures S10 and S11 and Table S11 show that the average slopes of the scaling relations are slightly different: 0.91 vs 0.81 in the right panel of Figure S10 for *NOH vs *NO and *NHO vs *NO, respectively. As a result, the lines intersect each other at rather negative adsorption energies ($\Delta G_{NO} < -3$ eV).

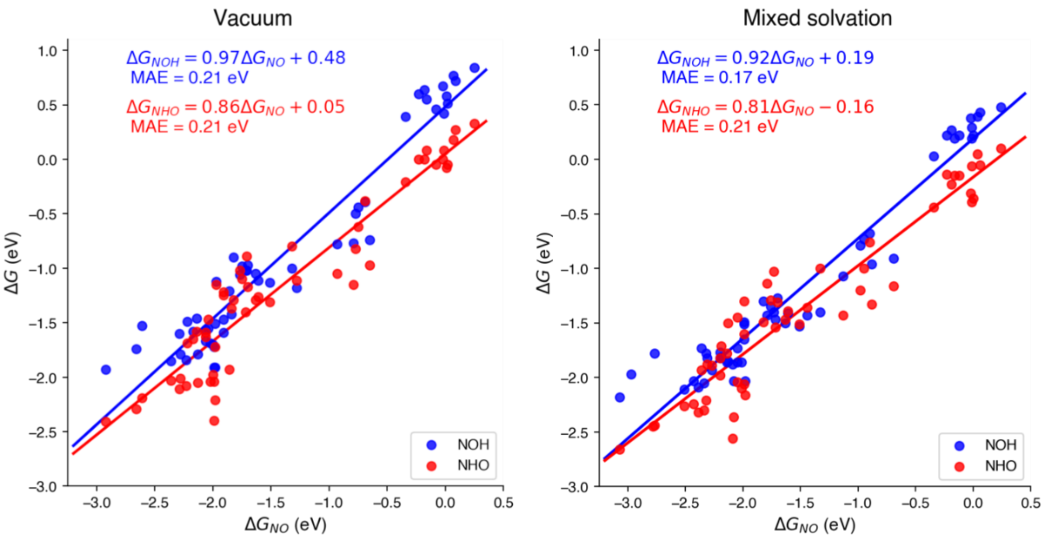

Figure S10. Scaling relations of ΔG_{NOH} *vs* ΔG_{NO} (blue) and ΔG_{NHO} *vs* ΔG_{NO} (red) on nine transition metals and six different surface sites from calculations in vacuum (left panel) and applying a mixed solvation model [4,5,7] (right panel). The free energies of adsorption are reported in Tables S9-S10.

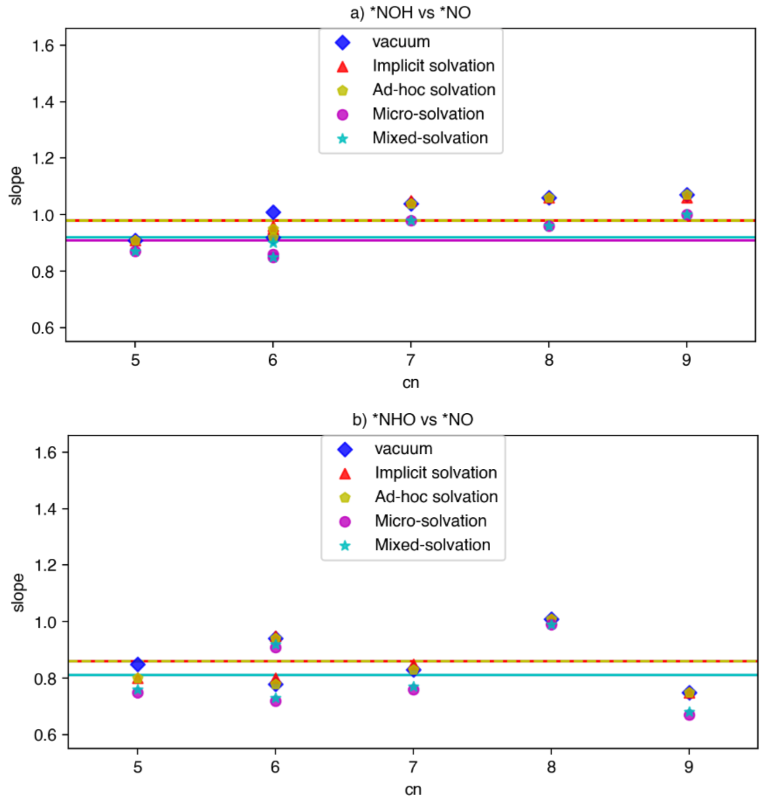

Figure S11. Slopes of the scaling relations as a function of the coordination number (*cn*) of the adsorption sites for: a) ΔG_{NOH} *vs* ΔG_{NO}, and b) ΔG_{NHO} *vs* ΔG_{NO} in vacuum and with different solvation models. The lines are the slopes of the scaling relations in Figure S10, obtained by plotting the data altogether (see specific values in Table S11). The small scattering with respect to the lines enable the use of a single linear fit for each dataset.

As numerous DFT studies on NO_x electroreduction represent polycrystalline fcc metal catalysts by considering just their (111) surface,[15,16] it is worth analyzing the scaling relations on that facet separately. Figure S12 shows that the slope of *NOH vs *NO on the (111) facet is close to the general one in Figure S10 (1.00 vs 0.91) for data with micro-solvation and a mixed solvation approach. However, this is not the case for *NHO vs *NO (0.67 vs 0.81). Figure S12 shows that these discrepancies persist when solvation is approximated in other ways or omitted. The result of the less steep slope for *NHO vs *NO is clear in Figure S12: the two lines intersect around $\Delta G_{NO} = -1.01 \pm 0.09$ eV, i.e., between Cu and Pt. This result is specific of the (111) facet, as the linear fits for ΔG_{NOH} *vs* ΔG_{NO} and ΔG_{NHO} *vs* ΔG_{NO} on all other facets do not intersect in the range of metals inspected, with *NOH lines typically above those of *NHO (see Figures S13-S17). This suggests that (111) terraces may not be representative of polycrystalline electrodes for *NO hydrogenation.

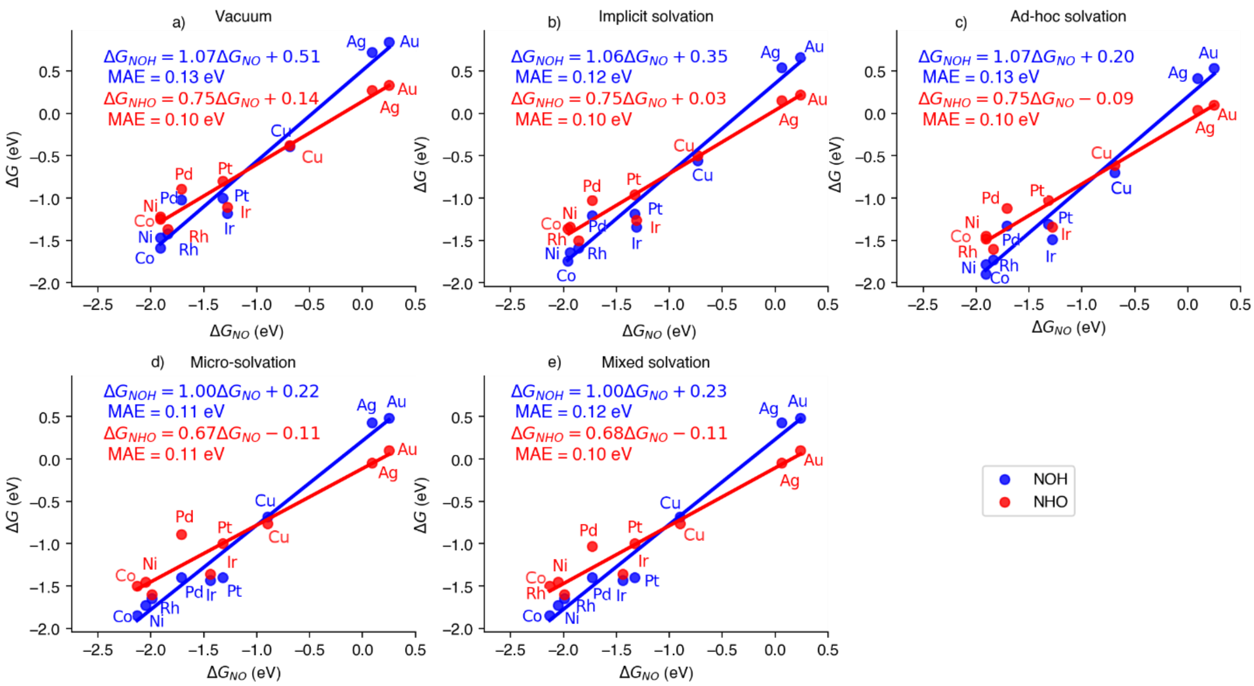

Figure S12. Scaling relations of ΔG_{NOH} *vs* ΔG_{NO} (blue) and ΔG_{NHO} (red) for the (111) facet of nine transition metals. a) In vacuum, b) with implicit solvation,[3,4] c) with ad-hoc solvation,[5] d) with micro-solvation,[6] e) with mixed solvation. The lines always intersect in the region between Cu and Pt (ΔG_{NO} = –1.01 ± 0.09 eV), such that Cu, Ag and Au produce *NHO, while strong-binding metals favor *NOH.

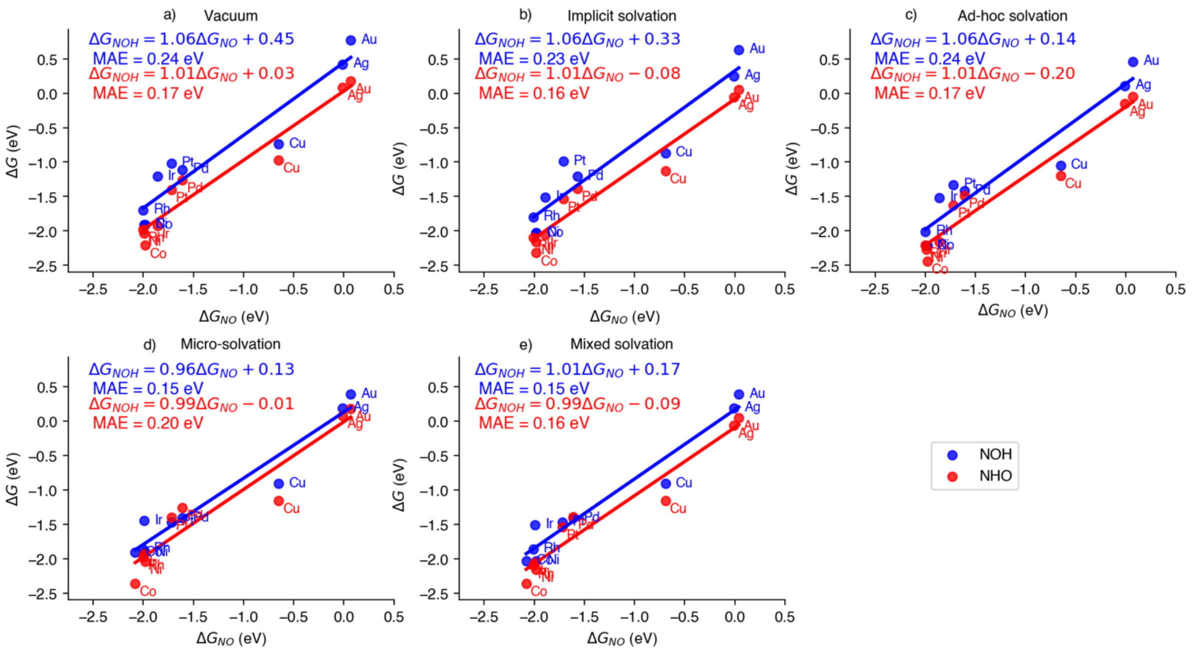

Figure S13. Scaling relations for the (100) facet in vacuum and with solvation corrections.

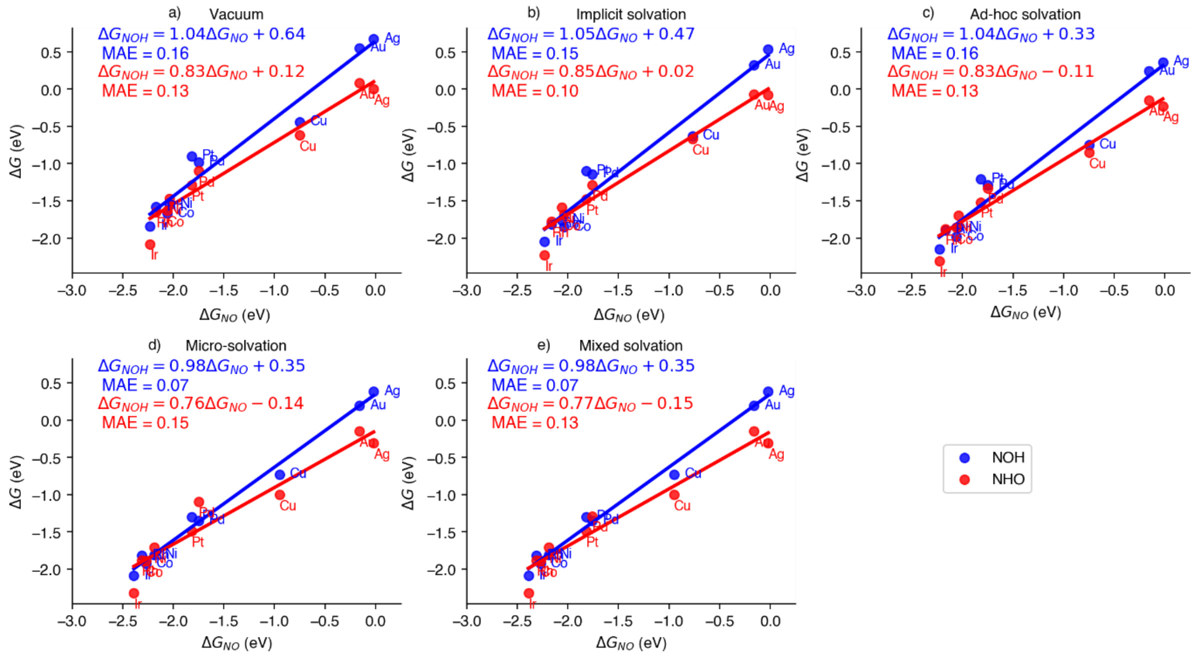

Figure S14. Scaling relations for the (211) facet in vacuum and with solvation corrections.

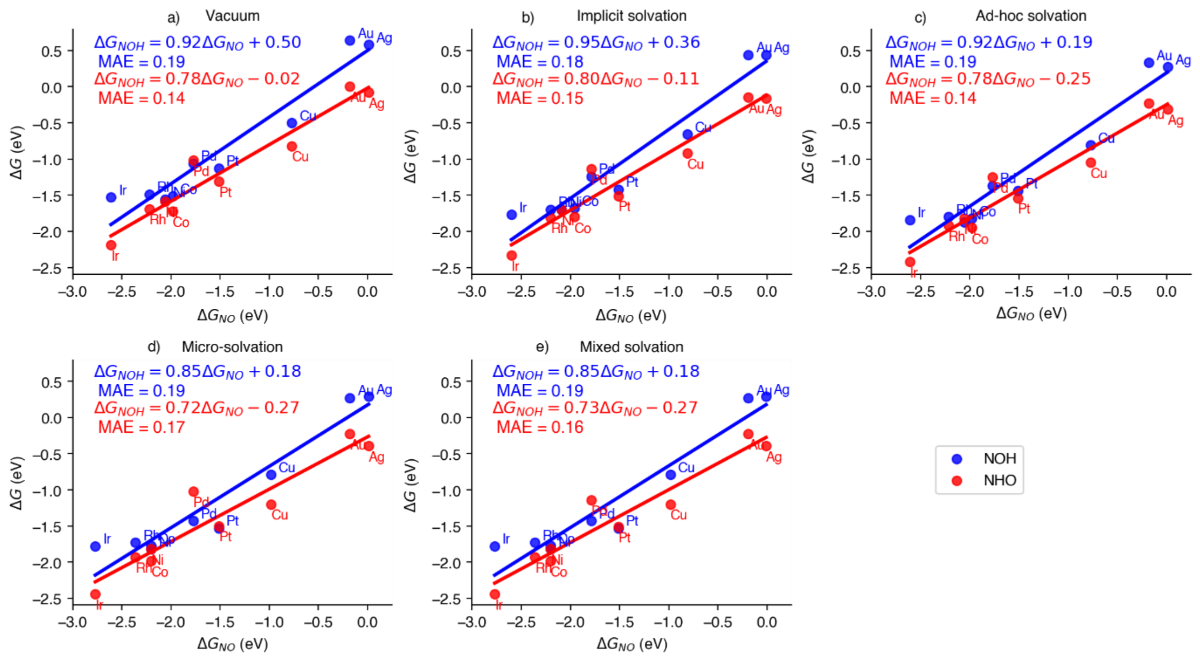

Figure S15. Scaling relations for the (211)k facet in vacuum and with solvation corrections.

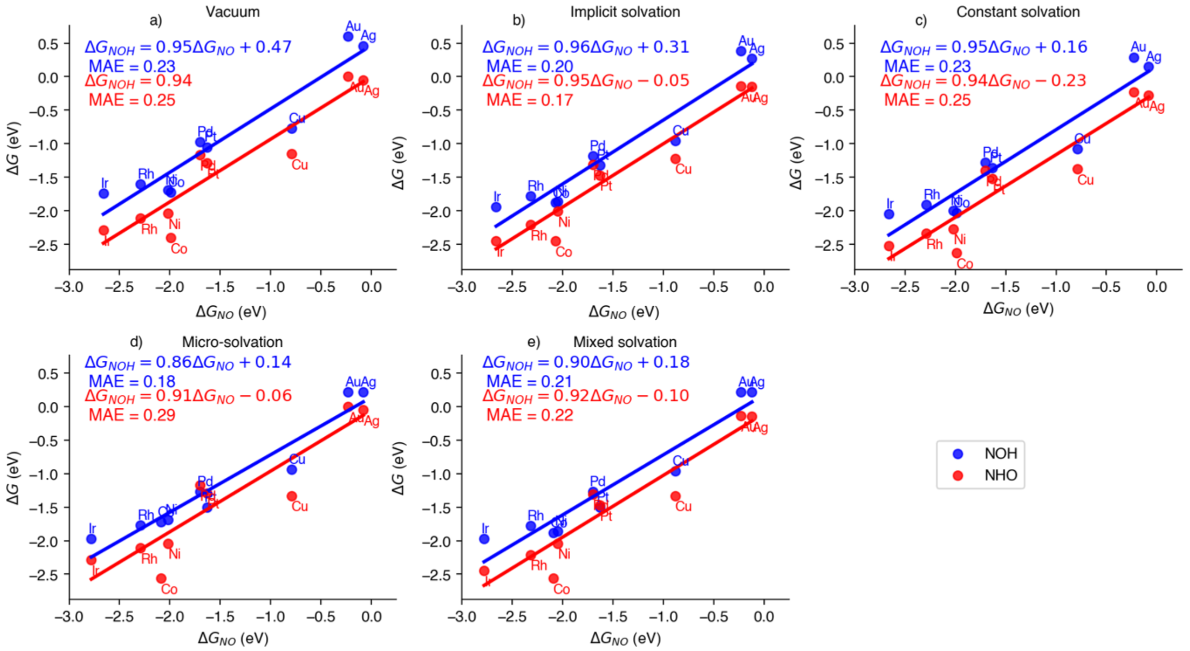

Figure S16. Scaling relations for the 4AD@(100) facet in vacuum and with solvation corrections.

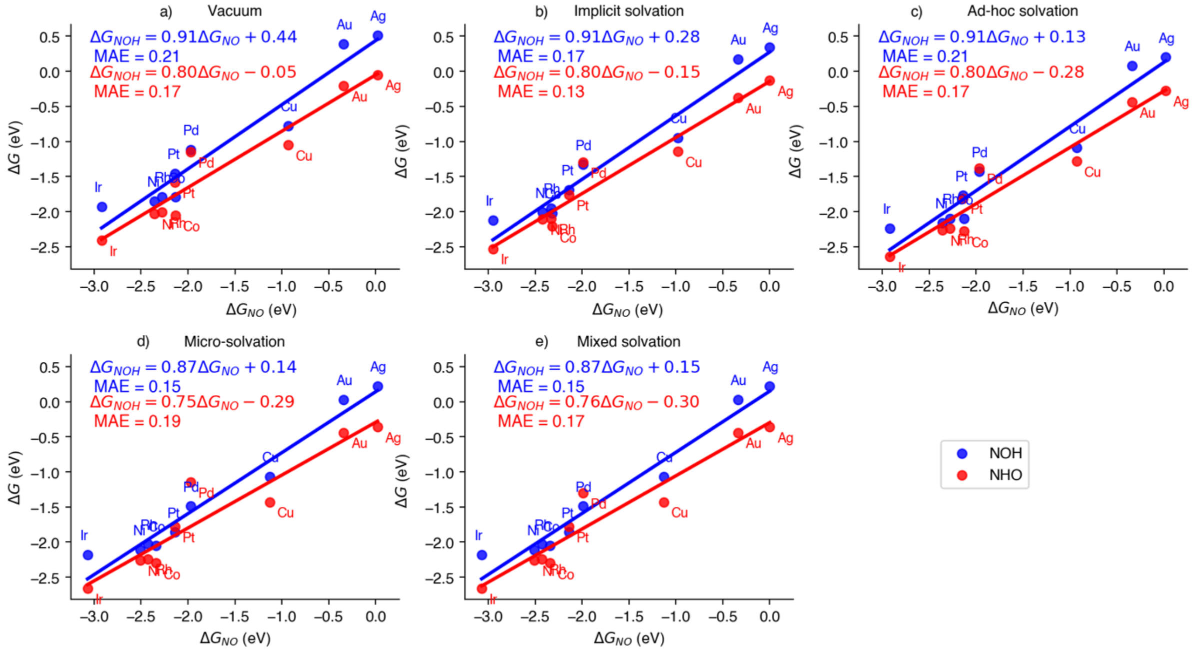

Figure S17. Scaling relations for the 3AD@(111) facet in vacuum and with solvation corrections.

S9. Slopes of the scaling relations

Table S11. Slopes of the scaling relations for ΔG_{NOH} *vs* ΔG_{NO} and ΔG_{NHO} *vs* ΔG_{NO} for model calculations in vacuum and with the different solvent models.

**ΔG_{NOH} *vs* ΔG_{NO}**						
**facet**	***cn***	**vacuum**	**implicit**	**ad hoc**	**micro-solvation**	**mixed**
all	–	0.97	0.98	0.97	0.90	0.92
111	9	1.07	1.06	1.07	1.00	1.00
100	8	1.06	1.06	1.06	0.96	1.01
211	7	1.04	1.05	1.04	0.98	0.98
211k	6	0.92	0.95	0.92	0.85	0.85
4AD @ 100	6	0.95	0.96	0.95	0.86	0.90
3AD @ 111	5	0.91	0.91	0.91	0.87	0.87
	avg	0.99	1.00	0.99	0.92	0.93
	std. dev.	0.07	0.07	0.07	0.07	0.07
**ΔG_{NHO} *vs* ΔG_{NO}**						
**facet**	***cn***	**vacuum**	**implicit**	**ad hoc**	**micro-solvation**	**mixed**
all	–	0.86	0.86	0.86	0.80	0.81
111	9	0.75	0.75	0.75	0.67	0.68
100	8	1.01	1.01	1.01	0.99	0.99
211	7	0.83	0.85	0.83	0.76	0.77
211k	6	0.78	0.80	0.78	0.72	0.73
4AD @ 100	6	0.94	0.95	0.94	0.91	0.92
3AD @ 111	5	0.85	0.80	0.80	0.75	0.76
	avg	0.84	0.86	0.85	0.80	0.81
	std. dev.	0.08	0.10	0.10	0.12	0.12

S10. Multivariate regressions

The multivariate regressions of the data were carried out using the “Analysis ToolPak” of MS Excel. In the y range we put the activity (A) classification based on the onset potential required for *NO hydrogenation. The classification distinguishes between very active ($U_{onset} > 0.3\ V$, yellow), active ($-0.05\ V < U_{onset} < 0.3\ V$, orange) and inactive sites ($U_{onset} < -0.05\ V$, red). Initially, the x range contains for each active site its coordination number (cn) and the group number (G) and period (P) of the metal in the periodic table. For instance, for 3AD@Cu(111) the values are, respectively, 5, 11, 4. The equation of the first multivariate regression is:

$$A = -1.3700 + 0.0080cn + 0.1485G - 0.0490P \qquad \text{(S12)}$$

The mean absolute error (MAE) between the regression and the calculated data for the onset potentials is 0.19 eV. The catalytic matrix predicted by means of the multivariate regression is provided in Table S12. The predicted matrix is able to locate a region of high

activity at group 11 elements (Cu, Ag, Au) and predicts that Ni sites are active, which is true in Table 3 for square sites. However, the regression does not predict that the (100) sites of group 9 metals be active. Quantitatively speaking, the regression is able to distinguish between active and inactive sites in 74% of the cases. Interestingly, the model has a probability of 89% of identifying an inactive site.

When an energy parameter is incorporated in the set of independent variables, the predictions improve, see Table 4 in the main text. In fact, the MAE is now 0.15 eV and more square sites at (100) and 4AD@(100) facets are predicted to be active. In quantitative terms, now the model correctly distinguishes between active and inactive sites in 87% of the cases. The equation of the multivariate regression is:

$$A = -1.2166 + 0.0489cn + 0.1061G - 0.0734P - 0.5741(\Delta G_{NHO} - \Delta G_{NOH}) \qquad \text{(S13)}$$

Table S12. Predicted activity matrix for *NO hydrogenation using as independent variables cn, G and P.

Surface	(111)	(100)	(211)	(211)k	4AD@(100)	3AD@(111)
cn	9	8	7	6	6	5
Co	inactive	inactive	inactive	inactive	inactive	inactive
Rh	inactive	inactive	inactive	inactive	inactive	inactive
Ir	inactive	inactive	inactive	inactive	inactive	inactive
Ni	active	active	active	active	active	active
Pd	inactive	inactive	inactive	inactive	inactive	inactive
Pt	inactive	inactive	inactive	inactive	inactive	inactive
Cu	active	active	active	active	active	active
Ag	active	active	active	active	active	active
Au	active	active	active	active	active	active

Now, because the onset potential is defined as: $U_{onset} \approx [\min(\Delta G_{*NOH}, \Delta G_{*NHO}) - \Delta G_{*NO}]/-1e^{-}$ and a large number of active sites produce *NHO from *NO (Table 2 in the main text), it is obvious that a regression including the adsorption energies of those two species should improve the predictiveness. In fact, the mean absolute error (MAE) of the multivariate regression based on coordination number, group number and the period of the metal in the

periodic table (cn, G, P) is 0.19 eV and goes down to 0.05 eV upon including ΔG_{*NHO} and ΔG_{*NO} as free parameters.

Our goal when using $\Delta G_{*NHO} - \Delta G_{*NOH}$ was not to use the most suitable energetic descriptor but rather one that is not directly related to U_{onset}. We note that the relatively large MAE of the correlation based on cn, G, P probably stems from the fact that it extends over a considerably narrower range of energies compared to U_{onset}. Indeed, the ratio of the two adsorption-energy spans is 0.44. This means that multivariate regressions based on cn, G, P can distinguish between the activity and inactivity regions of the matrices to some extent, but they are not able to predict any "very active" catalyst, as that category is located toward the right extreme of the energy range. When ΔG_{*NHO} and ΔG_{*NO} are added as free parameters, the span ratio goes to 1.04 and the predictions are rather similar to the computed activity matrices in Table 4, see Table S13 below. In turn, adding $\Delta G_{*NHO} - \Delta G_{*NOH}$ as a free parameter lowers the MAE to 0.15 eV and increases the span ratio to 0.75.

Table S13. Predicted activity matrix for *NO hydrogenation using as independent variables cn, G, P, ΔG_{*NHO} and ΔG_{*NO}.

Surface	(111)	(100)	(211)	(211)k	4AD@(100)	3AD@(111)
cn	9	8	7	6	6	5
Co	inactive	active	inactive	inactive	very active	inactive
Rh	inactive	active	inactive	inactive	inactive	inactive
Ir	active	active	inactive	inactive	inactive	inactive
Ni	inactive	active	inactive	inactive	active	inactive
Pd	inactive	inactive	inactive	inactive	inactive	inactive
Pt	inactive	inactive	inactive	active	inactive	inactive
Cu	active	very active	active	active	very active	active
Ag	active	active	active	very active	active	active
Au	active	active	active	active	inactive	active

Finally, the equation of the multivariate regression for the data in Table S13 is:

$$A = -0.0432 + 0.0196cn + 0.0044G - 0.0178P - 0.7767\Delta G_{*NHO} + 0.7955\Delta G_{*NO} \quad \text{(S14)}$$

S11. References

1 I. Katsounaros, M. C. Figueiredo, X. Chen, F. Calle-Vallejo and M. T. M. Koper, Structure- and Coverage-Sensitive Mechanism of NO Reduction on Platinum Electrodes, *ACS Catal.*, 2017, **7**, 4660–4667.

2 F. Calle-Vallejo and M. T. M. Koper, Accounting for Bifurcating Pathways in the Screening for CO2 Reduction Catalysts, *ACS Catal.*, 2017, **7**, 7346–7351.

3 H. J. Monkhorst and J. D. Pack, Special points for Brillouin-zone integrations, *Phys. Rev. B*, 1976, **13**, 5188.

4 K. Mathew, R. Sundararaman, K. Letchworth-Weaver, T. A. Arias and R. G. Hennig, Implicit solvation model for density-functional study of nanocrystal surfaces and reaction pathways, *J. Chem. Phys.*, 2014, **140**, 084106.

5 K. Mathew, V. S. C. Kolluru, S. Mula, S. N. Steinmann and R. G. Hennig, Implicit self-consistent electrolyte model in plane-wave density-functional theory, *J. Chem. Phys.*, 2019, **151**, 234101.

6 A. Clayborne, H.-J. Chun, R. B. Rankin and J. Greeley, Elucidation of Pathways for NO Electroreduction on Pt(111) from First Principles, *Angew. Chem. Internat. Ed.*, 2015, **54**, 8255–8258.

7 A. Rendón-Calle, S. Builes and F. Calle-Vallejo, Substantial improvement of electrocatalytic predictions by systematic assessment of solvent effects on adsorption energies, *Appl. Catal. B*, 2020, **276**, 119147.

8 M. T. M. Koper, Thermodynamic theory of multi-electron transfer reactions: Implications for electrocatalysis, *J. Electroanal. Chem.*, 2011, **660**, 254–260.

9 F. Calle-Vallejo, D. Loffreda, M. T. M. Koper and P. Sautet, Introducing structural sensitivity into adsorption–energy scaling relations by means of coordination numbers, *Nature Chem.*, 2015, **7**, 403–410.

10 M. J. Kolb, D. Loffreda, P. Sautet and F. Calle-Vallejo, Structure-sensitive scaling relations among carbon-containing species and their possible impact on CO2 electroreduction, *J. Catal.*, 2021, **395**, 136–142.

11 F. Calle-Vallejo, J. I. Martínez, J. M. García-Lastra, J. Rossmeisl and M. T. M. Koper, Physical and chemical nature of the scaling relations between adsorption energies of atoms on metal surfaces, *Phys. Rev. Lett.*, 2012, **108**, 116103.

12 H. Y. Su, K. Sun, W. Q. Wang, Z. Zeng, F. Calle-Vallejo and W. X. Li, Establishing and Understanding Adsorption-Energy Scaling Relations with Negative Slopes, *J. Phys. Chem. Lett.*, 2016, **7**, 5302–5306.

13 A. Brito-Ravicini and F. Calle-Vallejo, Interplaying coordination and ligand effects to break or make adsorption-energy scaling relations, *Exploration*, 2022, **2**, 20210062.

14 F. Calle-Vallejo, A. Krabbe and J. M. García-Lastra, How covalence breaks adsorption-energy scaling relations and solvation restores them, *Chem. Sci.*, 2016, **8**, 124–130.

15 H. Wan, A. Bagger and J. Rossmeisl, Electrochemical Nitric Oxide Reduction on Metal Surfaces, *Angew. Chem.*, 2021, **133**, 22137–22143.

16 C. A. Casey-Stevens, H. Ásmundsson, E. Skúlason and A. L. Garden, A density functional theory study of the mechanism and onset potentials for the major products of NO electroreduction on transition metal catalysts, *Appl. Surf. Sci.*, 2021, **552**, 149063.
